# AI-enabled workflow for automated classification and analysis of feto-placental Doppler images

**DOI:** 10.3389/fdgth.2024.1455767

**Published:** 2024-10-16

**Authors:** Ainhoa M. Aguado, Guillermo Jimenez-Perez, Devyani Chowdhury, Josa Prats-Valero, Sergio Sánchez-Martínez, Zahra Hoodbhoy, Shazia Mohsin, Roberta Castellani, Lea Testa, Fàtima Crispi, Bart Bijnens, Babar Hasan, Gabriel Bernardino

**Affiliations:** ^1^ BCN-MedTech, DTIC, Universitat Pompeu Fabra, Barcelona, Spain; ^2^ Institut d’Investigacions Biomèdiques August Pi I Sunyer (IDIBAPS), Barcelona, Spain; ^3^ Cardiology Care for Children, Lancaster, PA, United States; ^4^ Department of Paediatrics and Child Health, The Aga Khan University, Karachi, Pakistan; ^5^ Sindh Institute of Urology and Transplantation (SIUT), Karachi, Pakistan; ^6^ BCNatal—Barcelona Center for Maternal-Fetal and Neonatal Medicine (Hospital Clínic and Hospital Sant Joan de Déu), Universitat de Barcelona, Centre for Biomedical Research on Rare Diseases (CIBER-ER), Barcelona, Spain; ^7^ ICREA, Barcelona, Spain

**Keywords:** artificial intelligence, convolutional neural networks, deep learning, ultrasound view classification, ultrasound waveform delineation, feto-placental Doppler

## Abstract

**Introduction:**

Extraction of Doppler-based measurements from feto-placental Doppler images is crucial in identifying vulnerable new-borns prenatally. However, this process is time-consuming, operator dependent, and prone to errors.

**Methods:**

To address this, our study introduces an artificial intelligence (AI) enabled workflow for automating feto-placental Doppler measurements from four sites (i.e., Umbilical Artery (UA), Middle Cerebral Artery (MCA), Aortic Isthmus (AoI) and Left Ventricular Inflow and Outflow (LVIO)), involving classification and waveform delineation tasks. Derived from data from a low- and middle-income country, our approach's versatility was tested and validated using a dataset from a high-income country, showcasing its potential for standardized and accurate analysis across varied healthcare settings.

**Results:**

The classification of Doppler views was approached through three distinct blocks: (i) a Doppler velocity amplitude-based model with an accuracy of 94%, (ii) two Convolutional Neural Networks (CNN) with accuracies of 89.2% and 67.3%, and (iii) Doppler view- and dataset-dependent confidence models to detect misclassifications with an accuracy higher than 85%. The extraction of Doppler indices utilized Doppler-view dependent CNNs coupled with post-processing techniques. Results yielded a mean absolute percentage error of 6.1 ± 4.9% (*n* = 682), 1.8 ± 1.5% (*n* = 1,480), 4.7 ± 4.0% (*n* = 717), 3.5 ± 3.1% (*n* = 1,318) for the magnitude location of the systolic peak in LVIO, UA, AoI and MCA views, respectively.

**Conclusions:**

The developed models proved to be highly accurate in classifying Doppler views and extracting essential measurements from Doppler images. The integration of this AI-enabled workflow holds significant promise in reducing the manual workload and enhancing the efficiency of feto-placental Doppler image analysis, even for non-trained readers.

## 1 Introduction

Feto-placental Doppler imaging is the most widely used ultrasound technique for fetal health monitoring and assessment (1). In a non-invasive, quick, and secure manner, ultrasound is used to assess fetal and vascular development information and detect congenital heart diseases (CHD) (1). Doppler imaging allows a hemodynamic and physiological assessment of the cardiovascular system and fetoplacental circulation (2). Thanks to these advantages, and while the use of MRI is crucial to evaluate some particular conditions of the placenta and fetal 3D flow (3, 4), ultrasound remains the primary tool for the evaluation of fetal health as it allows quantifying the blood flow in critical regions like the umbilical artery (UA), middle cerebral artery (MCA), left ventricular inflow outflow (LVIO) and aortic isthmus (AoI). Measurements extracted from these regions have been shown to help identifying fetuses as Small Vulnerable Newborns (5, 6), a condition highly associated with neonatal death and morbidity.

**Figure 1:**
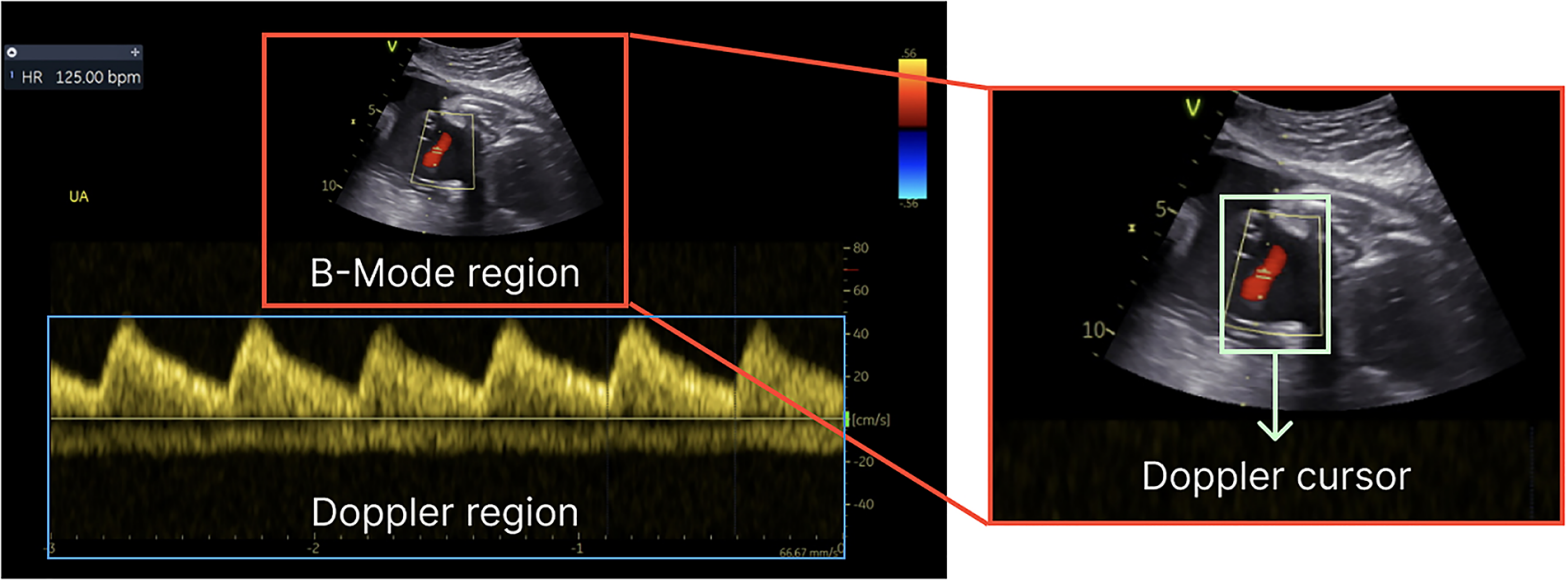
Components of a Doppler image—B-mode for structural details, the Doppler region for blood flow information and Doppler cursor on top of the B-mode region.

In a standard feto-placental Doppler study, a sequence of Doppler images is acquired over time and manually analyzed to evaluate fetal health. In addition to the Doppler spectrum, which represents the blood flow velocities over time (with the *x*-axis representing time and the y-axis showing the velocity of the blood flow), the Doppler images also include a brightness mode (B-mode or 2D) subimage. The subimage is employed to identify the specific spatial location from which the Doppler spectrum is obtained, as illustrated in Figure 1. It represents the anatomical structure at the designated time point and serves as a fixed reference point to facilitate visualization of the region where the Doppler measurements are being conducted. The acquisition time for these images is approximately 45–60 min; however, the subsequent manual analysis may span a longer period due to the following factors: (i) the large volume of images acquired, (ii) the unpredictable foetus positioning inside mother's womb, which increases image variability, and (iii) the numerous measurements required to be performed, as suggested by the ISUOG Practice guidelines (7). The analysis process consists of: (i) labelling each acquired image (classification), (ii) delineating its Doppler trace and (iii) retrieving functional Doppler indices crucial for clinical diagnosis (e.g., maximal peak velocity). Besides being a time-consuming task, this analysis heavily relies on operator's skill and often leads to inter- and intra-observational errors (1). For instance, a study published in 2013 by Vilkomerson et al. (8) reported a 25% inter-observer variability when measuring maximal peak velocity in Doppler images.

Artificial intelligence (AI), especially deep learning (DL), can be applied to the analysis of ultrasonographic studies, leading to faster and more standardised analysis of the acquired images as compared to manual analysis methods (9). In this sense, the manual steps requiring expertise could be replaced or supported by AI-enabled models specifically designed for labelling (10–13) or segmenting ultrasound images (14–18). Several studies, as the one exemplified by Gilbert et al. (12), have demonstrated the application of DL to automate the labelling process for ultrasonographic B-mode images achieving an accuracy of 87%–92% (11, 12). Other studies leveraged the potential of DL models for the classification of fetal ultrasound biometric images (i.e., abdomen, brain, thorax, and femur) (10, 13) demonstrating accuracies as high as 99.84%. Likewise, other studies have focused on automating segmentation and extracting measurements from echocardiographic images using AI (19, 20). In (19), correlation coefficients (0.988 and 0.985) between the automated and manual annotation of Mitral Valve inflow Doppler velocities were achieved, and in (18), the authors reported a bias of 0.31 cm/s and 0.14 cm/s, and standard deviations of 2.00 cm/s and 1.54 cm/s for the detection of the E and A peaks, respectively. Meanwhile, Marzbanrad et al. (20) reported a mean error of 15 ± 0.6 ms for the timing of the Aortic Valve outflow fetal cardiac intervals. Despite the remarkable acceleration in extracting information from the Doppler region facilitated by AI-enabled solutions, offering both high specificity and accuracy, it is important to note that several studies continue to rely on image processing techniques for doing so (21, 22), demonstrating agreement as high as (*R*^2^ = 0.94 and *R*^2^ = 0.90) between automated and manual measurements of peak velocity and Velocity Time Integral (VTI).

The aforementioned studies provide only partial solutions within the whole clinical pipeline, and very few of them focus on the development of such tools for fetal Doppler. To arrive at a documented interpretation of the Doppler image and a diagnosis, a sequence of tasks comprising view labelling, Doppler trace delineation, and automatic retrieval of Doppler-based imaging markers is essential. With these requirements in mind, this study presents the development of an AI-enabled pipeline that optimizes the clinical workflow in fetal echography, focused on the UA, MCA, AoI and LVIO. Notably, our AI models conforming this workflow will be trained using data from a Low-Middle Income Country (LMIC) and the generalisation and performance of the developed workflow will be reported against data from a high-income country.

## 2 Methodology

### 2.1 Dataset

The sample for this work comprised two research fetal ultrasound cohorts, which were acquired in accordance with the international guidelines for ultrasound velocimetry acquisition (7), with particular attention paid to mitigating the typical challenges associated with the acquisition of feto-placental Doppler images. Efforts were made to optimize the acquisition angle, to acquire multiple cardiac cycles (i.e., at least 2–3 cardiac cycles), to avoid aliasing and to account for fetal motion in order to minimize variability and maximize the quality of the images obtained.

The first one, FeDoC (Fetal Doppler Collaborative) (ClinicalTrials.gov Identifier: NCT03398551) study, was carried out on fetuses at the primary health care clinic operated by the Department of Paediatrics and Child Health at The Aga Khan University in Pakistan. The inclusion criteria specified pregnant women residing in the southeast region of Karachi (Pakistan) who were between 22 and 34 weeks of gestation and had provided written informed consent at the time of image acquisition (5). The images that conform this dataset were acquired using Vivid^TM^ iq (GE Healthcare, Zipf, Austria) Ultrasound System equipped with a curvilinear transducer.

The second one, sourced from the IMPACT trial (ClinicalTrials.gov Identifier: NCT03166332) from BCNatal-University of Barcelona (Spain), a randomized clinical trial that took place from 2017 to 2020 consisting of 1221 high-risk pregnant women. The echo images of this cohort were acquired using two different Ultrasound Systems from GE Healthcare: Voluson E10 and Voluson S8 (GE Healthcare, Zipf, Austria).

A total of 452 and 943 ultrasonographic studies were analysed, with 3,337 and 2,806 Doppler images, for FeDoC and IMPACT, respectively. These images were labelled and segmented (see Table 1) by a paediatric cardiologist and obstetrics fellow, for FeDoC and IMPACT, respectively, using an in-house cloud- and web-based platform: the *TransCor platform.* The *TransCor Platform* is a modular system developed at BCN-MedTech (Universitat Pompeu Fabra, Barcelona, Spain) and Insitut d'Investigacions Biomèdiques August Pi i Sunyer (IDIBAPS, Barcelona, Spain) consisting of several tools for ground-truth generation in Doppler images, such as view or anatomy labelling, cycle timing and Doppler waveform delineation based on the location of physiological events in the Doppler spectra (23, 24). For the ground-truth generation process, experts were first required to label each of the images by selecting the corresponding anatomy from a list of pre-defined fetal anatomies. Next, cardiac cycles needed to be delimited, due to the absence of ECG in feto-placental Doppler images, ejection beginning or beginning of systole events were used to define the start and the end of each of the cardiac cycles from the spectral Doppler. Experts were required to delimit a minimum of 2 cardiac cycles on each of the images. Lastly, experts were required to delineate the Doppler by locating a set of pre-defined anatomy-dependent physiological events, which were in turn used for generating a spline of the velocity envelope.

**Table 1:** Ground truth data generated using the *TransCor platform* for both datasets.

Label	FeDoC	IMPACT
Aortic Isthmus (AoI)	443	451
Middle Cerebral Artery (MCA)	424	428
Umbilical Artery (UA)	416	572
Left Ventricular Inflow Outflow (LVIO)	352	463
Other	1,702	892
Total	3,337	2,806

**Figure 2:**
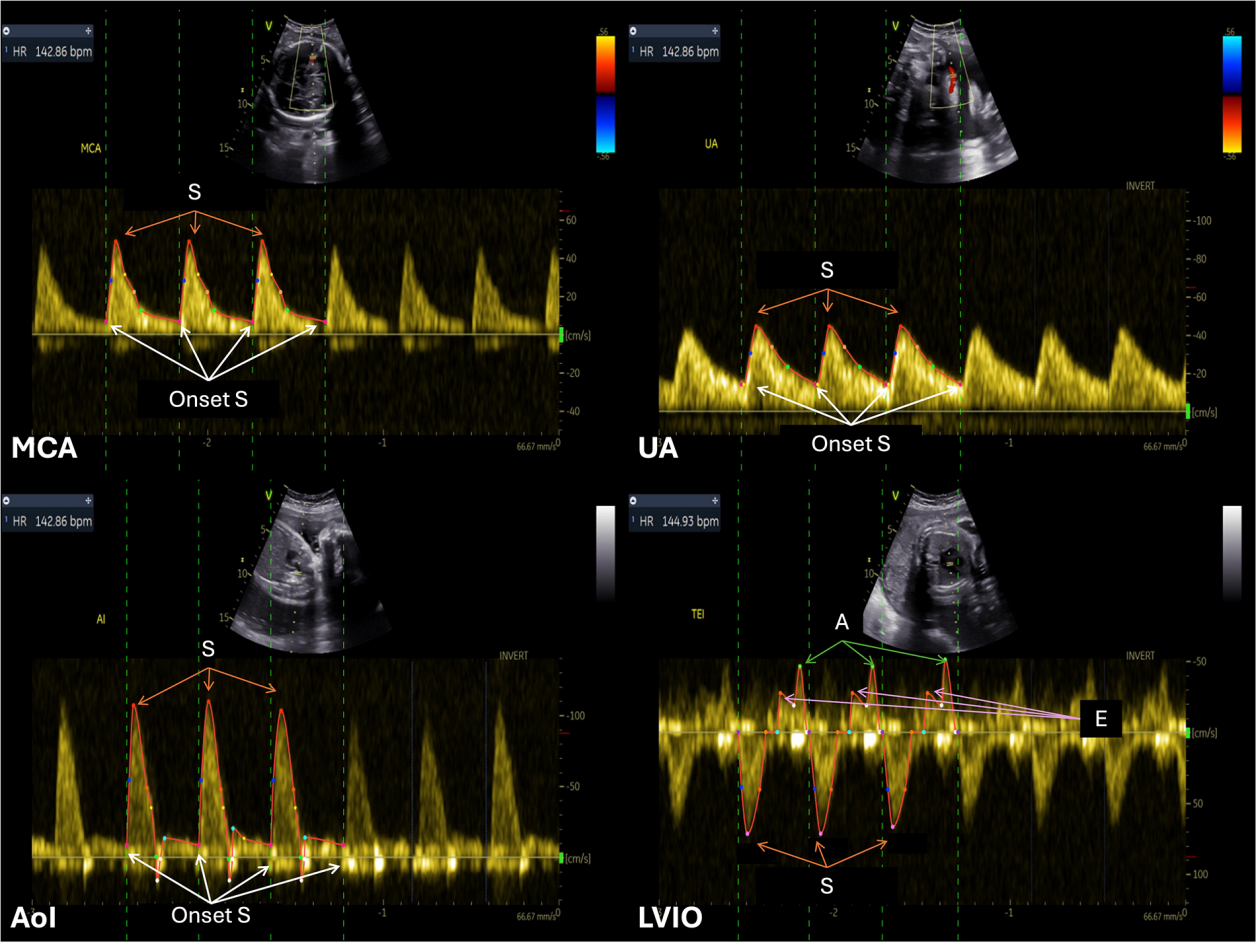
Example of annotated Doppler images from the feDoC dataset. The MCA, UA and AoI traces were delineated using the location of the S onset and the systolic peak value (S). The LVIO was traced with the S onset and systolic peak value to represent AV outflow and the E and A peaks were used for the MV inflow tracing.

This study focuses on four different feto-placental Doppler sites (see Figure 2): the MCA, UA, AoI, and LVIO. The MCA, focusing on cerebral circulation, was segmented by marking onset S and the systolic peak (S peak) in each cardiac cycle. Similarly, the UA, highlighting feto-placental blood flow, was traced using the same physiological events. Notably, both the MCA and UA Doppler signals consistently appeared either completely above or below the Doppler zero line. For the AoI, the key markers were placed at onset S and S peak. AoI images could include possible reversal flow, leading to the presence of Doppler signals on both sides of the zero line. Lastly, the LVIO captures combined blood flow through the mitral and aortic valves, which have opposite directions. This view was acquired using a cross-sectional view of the fetal thorax, at the level of the four-chamber view of the heart, a 2–4 mm Doppler sample volume was placed to include both the lateral wall of the ascending aorta and the mitral valve. The ventricular outflow tracing relied on systole (S) onset, S, and end of S, while the mitral inflow was traced through the onset of early diastolic (E) wave, E peak, atrial (A) peak and end of A wave. Besides the physiologically relevant control points mentioned, users could add additional control points to improve the fitting of a spline curve for defining the envelope.

We have included a detailed table in the (see Supplementary Table S1), which provides the mean, maximum and minimum durations of the Doppler spectrograms across both datasets.

### 2.2 Workflow description

**Figure 3:**
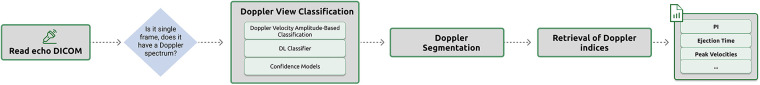
Schematic of the AI-enabled workflow for feto-placental Doppler, consisting of Doppler view classification and Doppler waveform delineation.

The proposed AI-enabled approach (see Figure 3) for automatically processing feto-placental Doppler ultrasound images involves two steps sequentially arranged. The first step is employed for view classification, which is identifying the specific Doppler view. The second step is the delineation of the temporal signal in the Doppler spectrum. For the scope of this study, only single frame fetal Doppler images were used, this was determined using the DICOM metadata.

For the first step, we used a sequential approach for view classification, consisting of three different blocks: (i) a Doppler amplitude-based classifier to divide the images based on the Doppler signal; (ii) DL-based classifiers that integrate B-mode and Doppler information; and (iii) confidence models that detect out-of-domain samples corresponding to other views. The classification task was divided into three blocks to overcome the limitations of previous attempts at using a single multiclass neural network. These attempts failed to distinguish between pulsatile and flatter Doppler signal profiles, as well as between the UA and MCA. The last block of the classifier, the confidence models, act as a quality control to detect and remove images corresponding to non-considered or non-standard views that might be included in a fetal study. The rationale for including it as a last step, rather than an initial one, is that the Doppler spectrograms can vary significantly depending on whether the view they represent is closer to the heart or further downstream. Additionally, integrating the different views can be challenging, particularly given the limited data available for analysis.

The objective of the second step, which comes after the view is classified in one of the pertinent classes using the models in the first step, is to extract the Doppler waveform by delineating its envelop, and the temporal localization of physiologically-relevant events (such as wave peaks). As the physiological events are dependent on the Doppler view, a waveform delineation model was created for each of the views. Based on the values of these events, we compute the clinically relevant Doppler indices used for medical assessment.

### 2.3 Pre-processing

B-Mode and spectral Doppler regions were identified from the DICOM file, using the publicly available metadata, and resized to 256 × 256 and 512 × 256, respectively.

DICOM images had cursor and burn-in annotations, which were in different vendor-specific colours. To standardise DICOM images, burned-in annotations were detected and removed, as well as the fully black rows and columns.

Additionally, the position of the Doppler cursor on top of the B-mode region was extracted by image processing. This step involved the detection of non-grey pixels within the image. The determined ROI location was then employed to generate a binary mask, which was subsequently combined with the B-mode image. This pre-processing step was essential due to the absence of Doppler cursor position in the publicly available metadata of the DICOM images. It should be noted, however, that the position of the Doppler cursor varies depending on the specific spectral Doppler modality used. It can be represented either as a dashed line, as shown in Supplementary Figure S14, or as a bounding box, as illustrated in Supplementary Figure S13.

Furthermore, to extract multiple metrics from the Doppler spectra, the Doppler region was binarized using simple thresholding. This process aimed to extract multiple metrics, such as the maximum (*V_max_*) and minimum (*V_min_*) velocities between positive and negative peaks, as well as their combined sum, termed as *V_range_*. The detection of positive and negative peaks was particularly advantageous for cropping this region to emphasise the Doppler signal.

**Figure 4:**
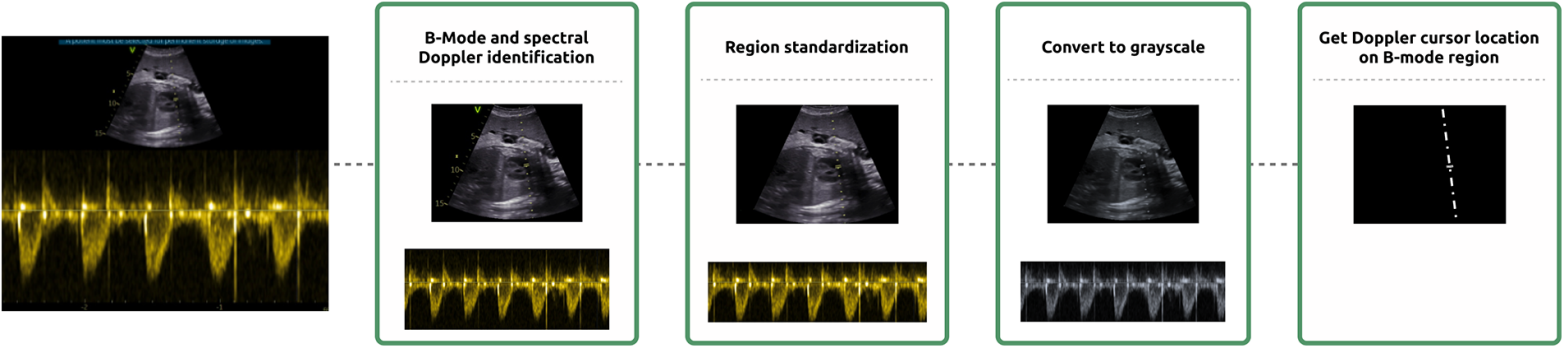
Schematization of the pre-processing steps. Pre-processing consisted of the identification of the B-Mode and spectral Doppler regions, region standardization with the removal of burnt-in annotation and cropping, grayscale conversion and the location of the Doppler cursor on top of the B-mode region.

Finally, both regions were converted to grayscale and its intensities normalized to [0, 1] range. A schematic representation of the pre-processing steps is provided in Figure 4.

### 2.4 Classifier

Leveraging physiological differences, the first step of the classifier consisted in grouping peripheral (UA and MCA) and cardiac/aortic Doppler patterns using a Doppler velocity amplitude-based classifier. This approach led to grouping the MCA and UA, distinguished by Doppler signals completely positioned entirely above or below the Doppler zero line, depending on the relative orientation of the probe. Conversely, the AoI and LVIO were also grouped. Therefore, the initial phase of classification aimed to distinguish between these two different Doppler patterns. To achieve this, a Doppler amplitude-based classifier was developed using classical machine learning models, to differentiate between images corresponding to the MCA-UA or AoI-LVIO groups. The K-Nearest Neighbours algorithm, with K = 13, was employed with the previously extracted *V_min_* and *V_range_* values. This methodology was designed to effectively categorise Doppler regions according to their analogous Doppler patterns and to redirect the DICOM image to the corresponding DL-based classifier.

Additionally, two DL-based classifiers were developed aimed to distinguish between: (i) MCA and UA, and (ii) LVIO and AoI. These classifiers made use of the information found in both, the Doppler spectra, and the B-mode preview. The chosen architecture for doing so was a parallel ResNet50 (25). This architecture consisted of two convolutional encoders from ResNet50, one for the B-mode and the other for the Doppler spectra, whose weights were initialised with an in-house paediatric Doppler classification model. The feature vectors generated from each encoder were concatenated to create a joint low-dimensional embedding. This embedding was then passed through a fully connected network with 3 layers: 2,048, 256, and 2 neurons. The weights were not shared between the two convolutional encoders in the model.

Furthermore, Doppler view and dataset dependent confidence models were developed to identify and discard images corresponding to other anatomic views not considered in this study (26, 27). These models used the DL description vectors generated from the ResNet50 with the default ImageNet weights (25) inputting either the Doppler or the B-mode region. The dimensionality of the resulting feature vectors underwent reduction through Principal Component Analysis, retaining features that accounted for 85% of the variance. Subsequently, these reduced-feature vectors were used to train individual XGBoost models (28) for each Doppler view and dataset. These models were trained using images from the class of interest, as well as those depicting other fetal cardiovascular structures or heart valves (i.e., tricuspid inflow, pulmonary artery). The selection of images for each model was guided by predictions from preceding models in the pipeline, the Doppler amplitude-based model, and the corresponding DL-based classification model. Any image predicted as the class of interest but actually not belonging to that class was retained and labelled under the “*skip*” category.

### 2.5 Doppler waveform delineation and physiological event detection

The selected architecture was the W-Net, chosen for its demonstrated success in various segmentation domains (29–31). The W-Net architecture consists of two stacked U-Net architectures, where the output of the first U-Net serves as the input to the second U-Net. To prevent a bottleneck between the two U-Nets, skip connections are employed between the decoder of the first U-Net and the encoder of the second U-Net, like the connections established between the encoder and decoder in a traditional U-Net. This additional structure increases the depth of the network, which often leads to improved performance (32). Notably, our approach diverged from the typical single-output structure by incorporating multiple output channels in the final layer of the W-Net: the first channel included the velocity envelope, and the remaining included the time-location of the physiological events. This architecture allowed our model to simultaneously delineate the Doppler envelope waveform and precisely locate targeted physiological events within it.

**Figure 5:**
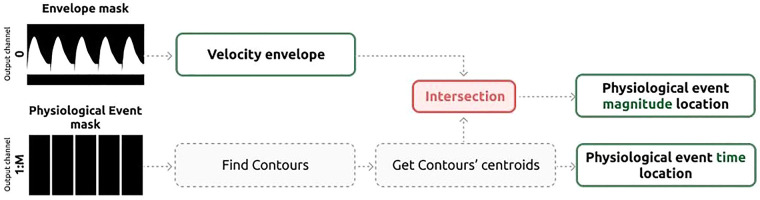
Schematic representation of the post-processing of the waveform delineation results to obtain the location in time and magnitude of the relevant physiological events.

After acquiring the binary masks representing the velocity envelope and the time-coordinates of targeted physiological events, our methodology involved a sequence of post-processing procedures (represented in Figure 5). These steps aimed to precisely determine both the temporal position and magnitude of the desired events within the Doppler signal, such as ejection beginning or peak velocity.

The initial step is to smooth the predicted binary mask corresponding to the Doppler envelope using a 7 × 7 Gaussian kernel, followed by binary dilation and closing. Subsequently, we determine the region in which the maximum absolute velocity is located—either below or above the Doppler zero line—and reset the values in the opposite region to zero. It's worth noting that for the LVIO Doppler images, this step is performed twice: once for the outflow pattern and once for the corresponding inflow pattern.

Next, the channels associated with desired physiological events undergo post-processing to precisely determine the *time*-coordinates of the detected events within the Doppler region. To begin, gaps are filled, and subsequently, the centroid of each onset is identified, establishing the *time*-coordinate of the control point. Detected *time*-coordinates are then forced to have a minimum distance of 80 ms between each other, following the approach described by Wong et al. (33). The enforced minimum distance between events of 80 ms was enforced, to avoid the detection of double peaks as shown in Supplementary Figure S15, and it was chosen empirically, based on the time-distance between the different physiological events.

Finally, the *magnitude*-coordinates for control points are derived by calculating the intersection between the previously determined *time*-coordinate of the event and the velocity envelope.

### 2.6 Training

Both classification and waveform delineation models were trained using a data augmentation approach. This involved concatenating the previously manually segmented cardiac cycles while modifying their duration and magnitude by a random factor up to 10%. These synthetic images were generated from annotated data and encompassed 1 to 18 cardiac cycles, with the definition of cardiac cycle numbers being randomized for each image. This strategy ensures the network's adaptability to spectrograms featuring varying numbers of cardiac cycles. This approach is particularly advantageous in real-world clinical scenarios where obtaining a predetermined number of cardiac cycles can be challenging due to factors such as fetal movement during image acquisition. In addition, each image in the Doppler waveform delineation model training set had two synthetic versions: one unchanged and another vertically flipped.

The FeDoC dataset served as the training data for each of the DL models presented in this study. For both classification and waveform delineation models, the dataset underwent a division into training (75%) and testing (25%) sets, ensuring a stratified split by class to maintain view distribution in both training and testing sets. In addition, images from the same patient were assigned either to the training or the testing dataset.

For the training of the Doppler waveform delineation models, the ground-truth generated by the two experts required to be transformed into binary masks. Consequently, the waveform delineation details for each of the Doppler images were encoded through a binary mask with *M* channels, aligning with the dimensions of the Doppler region within the image. Here, *M* signifies the count of intended physiological events for detection, along with an additional channel dedicated to the velocity envelope mask. Specifically, the values for *M* were 2, 2, 2, and 7 for AoI, MCA, UA, and LVIO, respectively.

In the DL models training phase, a batch size of 16 was used over 200 epochs and 100 epochs, for the DL-based classifiers and waveform delineation models, respectively.

Diverse augmentation techniques were applied at each epoch, affecting either the Doppler or B-mode regions of the image. The applied augmentation techniques included brightness and contrast adjustments, geometric transformations (e.g., flipping, rotation, scale), and custom functions tailored to address our specific problem, such as eliminating unnecessary rows in the Doppler region, simulating aliasing, and B-mode rotation. These augmentation techniques were systematically applied at each epoch, enriching the dataset, and enhancing the model's learning process. Optimization was achieved with the Adam optimizer and a learning rate of 1e-3. The learning process was finetuned using a *ReduceLROnPlateau* scheduler (34), which dynamically adjusted the learning rate by a factor of 0.1 after a patience of 20 epochs. The loss function employed for classification was cross-entropy, while for waveform delineation, both the Dice coefficient, measuring overlap between predicted and ground-truth masks, and the F1 loss, balancing specificity and sensitivity when measuring waveform delineation accuracy, were employed.

### 2.7 Evaluation

All generated models were evaluated on a separate test set from the FeDoC dataset, as well as the IMPACT dataset. Classification models were evaluated using standard classification metrics such as accuracy, specificity, sensitivity, F1 score and Area Under the Curve (AUC). The AUC was calculated by measuring the area under the curve, which was based on the prediction scores of each classification model (35).

The evaluation of the waveform delineation of UA, MCA and AoI was based on the pulsatility index (*PI*) (see Equation 1) and the estimation of the maximum and minimum velocities. Whereas LVIO's waveform delineation performance was based on the systolic (*S*) and diastolic (*D*) duration (Equation 2), velocity magnitudes of the S, E and A peaks. S duration was computed as the difference between the valve opening (*VO_t_*) and closure times (*VC_t_*), while the D duration was the difference between the end of the A wave (*end A_t_*) and the start of E (*onset E_t_*). The metrics were calculated as follows:

(1) $$PI=\frac{V_{\max}-V_{\min}}{V_{\mathrm{mean}}}$$

(2) $$\begin{array}{rl} S\;\mathrm{duration}\; & =VO_{t}-VC_{t},\\ D\;\mathrm{duration}\; & =\;\mathrm{end}\;A_{t}-{\;\mathrm{onset}\;E}_{t} \end{array}$$

The disparities between the ground-truth and the inferred annotations were reported in the form of Root Mean Square Errors (RMSE) or Mean Absolute Percentage Errors (MAPE). Furthermore, the RMSE and MAPE were calculated based on the median across all manually delineated cardiac cycles for a given image.

## 3 Results

**Figure 6:**
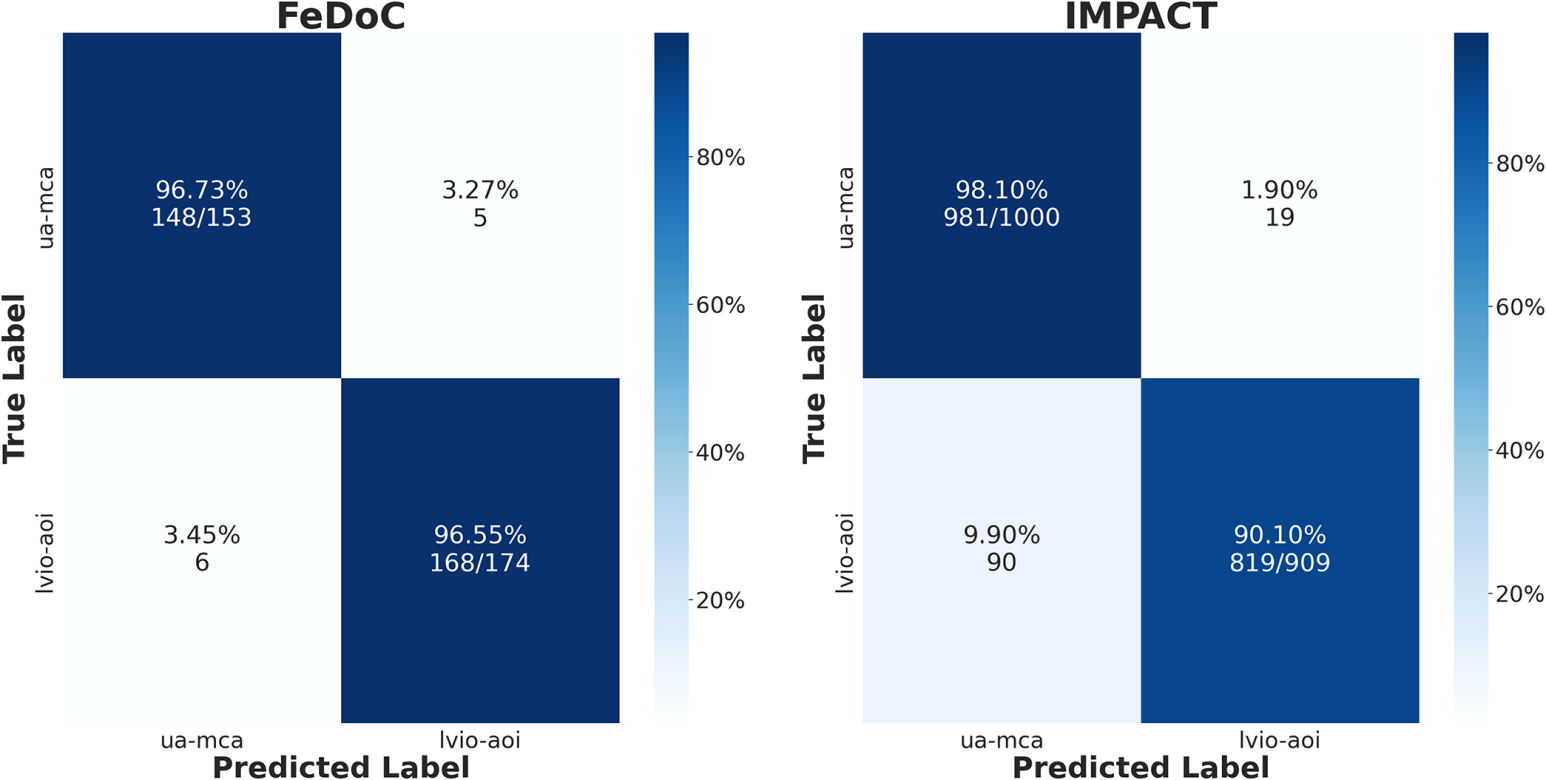
Confusion matrices of the classical machine learning model employed to distinguish between AoI-LVIO and UA-MCA using Doppler velocity ranges trained in the feDoC dataset.

The Doppler velocity amplitude-based classifier, employed to distinguish between the MCA-UA and LVIO-AoI using metrics extracted from the Doppler region, the *V_min_* and *V_range_*, presented an accuracy of 97% and 94% in FeDoC and IMPACT datasets, respectively (see Figure 6; Table 2).

**Table 2:** Doppler amplitude-based classification results.

	FeDoC—accuracy 97%				IMPACT—accuracy 94%			
Class	Specificity	Sensitivity	F1-score	Samples	Specificity	Sensitivity	F1-score	Samples
UA—MCA	0.97	0.97	0.96	153	0.98	0.91	0.95	1,000
AoI—LVIO	0.97	0.97	0.97	174	0.91	0.98	0.94	909
Macro Avg	0.97	0.97	0.97	327	0.94	0.94	0.94	1,909
Weighted Avg	0.97	0.97	0.97	327	0.94	0.94	0.94	1,909

In Table 3, the classical classification metrics for the Doppler view classification models are presented. An accuracy of 87.4% and 89.2% for the DL classification model aimed at distinguishing UA and MCA, in the FeDoC and IMPACT datasets respectively, was obtained. Conversely, the model aimed at distinguishing between the AoI and LVIO Doppler views demonstrated lower accuracies in IMPACT dataset, achieving 99.4% and 67.3% accuracy in the FeDoC and IMPACT datasets, respectively.

**Table 3:** Doppler view classification results for the UA-MCA classifier and AoI-LVIO classifier with no Doppler cursors.

UA vs. MCA								
Class	FeDoC—accuracy 87.4%				IMPACT—accuracy 89.2%			
	Specificity	Sensitivity	F1-score	Samples	Specificity	Sensitivity	F1-score	Samples
MCA	1.0	0.71	0.83	77	0.91	0.88	0.89	425
UA	0.71	1.0	0.89	98	0.88	0.91	0.89	425
Macro avg	0.86	0.86	0.87	175	0.89	0.89	0.89	850
Weighted Avg	0.85	0.87	0.87	175	0.89	0.89	0.89	850
AoI vs. LVIO								
Class	FeDoC—accuracy 99.4%				IMPACT—accuracy 67.3%			
	Specificity	Sensitivity	F1-score	Samples	Specificity	Sensitivity	F1-score	Samples
AoI	0.99	1.0	0.99	85	0.76	0.58	0.64	447
LVIO	1.0	0.99	0.99	88	0.58	0.76	0.69	447
Macro Avg	0.94	0.99	0.99	173	0.67	0.67	0.67	895
Weighted Avg	0.94	0.99	0.99	173	0.67	0.67	0.67	895

The Doppler view and dataset dependent confidence models, used to eliminate misclassified samples, demonstrated false negative rates (FNR) of less than 13% for all samples across the UA, MCA and AoI views in both datasets. In contrast, the FNR of the LVIO was 72.3%, as presented in Table 4. However, the False Positive Rate (FPR) was relatively low at 1.72%. Therefore, the model demonstrated a lack of efficacy in retaining samples for their subsequent analysis.

**Table 4:** Doppler view-dependent confidence models showing skip and keep accuracies for each view/label, false negative rata (FNR,%) and false positive rate (FPR,%) and the area under the curve (AUC).

Label	Dataset	FNR,%	FPR,%	AUC	Skip samples	Keep samples	Total samples
MCA	FeDoC	12.50	0	0.99	106	24	130
	IMPACT	4.81	7.52	0.98	266	208	474
UA	FeDoC	3.85	0.75	1	133	26	159
	IMPACT	9.49	0.92	1	977	253	1,230
AoI	FeDoC	3.77	2.49	1	241	106	347
	IMPACT	8.44	4.36	0.99	574	154	728
LVIO	FeDoC	72.15	1.72	0.87	524	79	603
	IMPACT	9.68	2.51	0.99	798	124	922

In the evaluation of the UA, MCA and AoI's waveform delineation models, the magnitude of the maximal (*V_max_*) and minimal velocity (*V_min_*), as well as the PI were assessed. The results, as detailed in Table 5, showcase notable variations in *V_max_*, *V_min_*, and PI throughout the two datasets. For instance, the *V_max_* in the UA presented of MAPE 2.7% and 1.8%, in FeDoC and IMPACT, respectively. The most challenging view across all Doppler-derived magnitudes was the MCA, which presented a maximum MAPE of 10.4 ± 7.0% and 12.1 ± 9.6% in the estimation of *V_max_* and *V_min_* in the FeDoC dataset. Regarding the timing of events and the duration of the cardiac cycles, detailed in Table 5, the smallest RMSEs were observed in the time location of peak velocities [*Peak*(*t*)]. The biggest error in peak velocity timing [*Peak*(*t*)] was found in the location of AoI's peak velocity in the FeDoC dataset, showing a RMSE of 8.1 ± 10.3 ms.

**Table 5:** Comparison of mean errors in inferred Doppler indices for MCA, UA and AoI among the feDoC and IMPACT datasets: pulsatility Index (PI), Maximum velocity (V_max_), and Minimum velocity (V_min_) reported as MAPE and RMSE, and timing of relevant physiological events and cardiac cycle curation, reported as RMSE.

Metric	Dataset	MCA	UA	AoI
V_max_,%	FeDoC	10.4 ± 7.0 (*n* = 75)	2.7 ± 2.6(*n* = 150)	6.1 ± 6.2 (*n* = 49)
	IMPACT	3.5 ± 3.1 (*n* = 1,318)	1.8 ± 1.5 (*n* = 1,480)	4.7 ± 4.0 (*n* = 717)
V_max_, cm/s	FeDoC	4.5 ± 4.9(*n* = 75)	1.9 ± 2.9(*n* = 150)	8.6 ± 13.1(*n* = 49)
	IMPACT	2.0 ± 3.0(*n* = 1,318)	1.0 ± 1.5 (*n* = 1,480)	5.0 ± 7.2 (*n* = 717)
V_min_,%	FeDoC	12.1 ± 9.6 (*n* = 76)	4.6 ± 4.0(*n* = 146)	8.5 ± 7.3 (*n* = 50)
	IMPACT	9.5 ± 6.5 (*n* = 1,281)	5.1 ± 4.0 (*n* = 1,475)	8.9 ± 8.8 (*n* = 710)
V_min_, cm/s	FeDoC	1.6 ± 2.1 (*n* = 76)	1.3 ± 1.6(*n* = 146)	1.4 ± 1.7 (*n* = 50)
	IMPACT	1.2 ± 1.4 (*n* = 1,281)	1.3 ± 1.4 (*n* = 1,475)	1.3 ± 2.6 (*n* = 710)
PI,%	FeDoC	10.06 ± 9.41 (*n* = 71)	4.67 ± 3.38 (*n* = 143)	5.44 ± 4.36 (*n* = 51)
	IMPACT	5.01 ± 3.81 (*n* = 1,329)	6.59 ± 4.37 (*n* = 1,483)	5.06 ± 8.71 (*n* = 760)
PI	FeDoC	0.16 ± 0.24 (*n* = 71)	0.04 ± 0.05 (*n* = 143)	0.16 ± 0.21 (*n* = 51)
	IMPACT	0.1 ± 0.12 (*n* = 1,329)	0.06 ± 0.08 (*n* = 1,483)	0.16 ± 0.36 (*n* = 760)
Onset S (t), ms	FeDoC	10.9 ± 9.4 (*n* = 56)	10.7 ± 10.2 (*n* = 121)	12.8 ± 10.7 (*n* = 37)
	IMPACT	8.2 ± 8.8 (*n* = 237)	7.2 ± 8.8 (*n* = 300)	10.6 ± 18.9 (*n* = 107)
Peak (t), ms	FeDoC	7.0 ± 7.8 (*n* = 56)	2.3 ± 3.0(*n* = 121)	8.1 ± 10.3 (*n* = 35)
	IMPACT	4.2 ± 7.1(*n* = 234)	1.4 ± 1.6 (*n* = 299)	3.2 ± 4.2 (*n* = 106)
Cycle length (t), ms	FeDoC	7.5 ± 7.4 (*n* = 51)	8.8 ± 8.1 (*n* = 114)	8.0 ± 8.1 (*n* = 31)
	IMPACT	9.7 ± 9.5 (*n* = 224)	8.8 ± 8.4 (*n* = 288)	8.3 ± 7.9 (*n* = 91)

In Table 6, errors pertaining to the timing location and magnitudes of the relevant physiological events (*S*, *E* and *A* peaks), along with the duration of the systolic and diastolic phases for LVIO, are presented. A MAPE of 6.1 ± 3.56% was reported in the peak velocity value of the systolic phase (*S*) for both datasets, while the error in the duration of the systolic phase was less than 15 ms. The observed MAPE errors in the location of *E* and *A* peaks were higher in the IMPACT dataset with 29.9 ± 5.7% and 12.6 ± 5.1%, respectively.

**Table 6:** Errors at the computation of clinical parameters for the left ventricular inflow outflow (LVIO) Doppler images.

Metric	Dataset	LVIO
S,%	FeDoC	6.1 ± 3.56 (*n* = 341)
	IMPACT	6.1 ± 4.9 (*n* = 682)
S, cm/s	FeDoC	6.1 ± 3.8 (*n* = 398)
	IMPACT	4.6 ± 2.8 (*n* = 682)
E,%	FeDoC	10.3 ± 3.5 (*n* = 370)
	IMPACT	29.9 ± 5.7 (*n* = 254)
E, cm/s	FeDoC	4.4 ± 3.0 (*n* = 370)
	IMPACT	11.4 ± 5.7 (*n* = 254)
A,%	FeDoC	9.7 ± 4.9 (*n* = 370)
	IMPACT	12.6 ± 5.1 (*n* = 262)
A, cm/s	FeDoC	5.7 ± 3.9 (*n* = 370)
	IMPACT	7.3 ± 4.9 (*n* = 262)
S (t), ms	FeDoC	10.0 ± 9.101 (*n* = 200)
	IMPACT	9.5 ± 9.1 (*n* = 349)
E (t), ms	FeDoC	13.6 ± 11.192 (*n* = 192)
	IMPACT	32.1 ± 22.5 (*n* = 204)
A (t), ms	FeDoC	18.7 ± 17.6 (*n* = 197)
	IMPACT	24.0 ± 19.8 (*n* = 145)
S duration (t), ms	FeDoC	11.8 ± 7.3 (*n* = 380)
	IMPACT	14.8 ± 8.2 (*n* = 356)
D duration (t), ms	FeDoC	14.9 ± 9.2 (*n* = 380)
	IMPACT	17.9 ± 10.9 (*n* = 352)

Metrics that include (t) denote timings (x axis); their errors are the root mean square error (RMSE) in milliseconds. Metrics that do not include (t) denote amplitudes (y axis) and their errors are the RMSE and Mean Average Percentage Error (MAPE).

## 4 Discussion

In this study, an AI-enabled workflow for automatically quantifying feto-placental Doppler images was developed. The results demonstrate the potential of using AI models to optimize fetal Doppler analysis by replacing time-consuming tasks such as manual image classification and identification of physiological events in the Doppler region by DL-based methods, thereby reducing analysis time for each ultrasonographic study.

Automated analysis of Doppler spectrograms, despite its clinical relevance, has been overlooked (36). There exist only few works, focusing on adult applications. Despite the velocity waveform being a time signal, authors have taken an approach based on 2D image processing, which allows to take advantage of contextual information (i.e., pixels that are both close in time (*x*-axis), but also in velocity (y-axis)) (14, 18, 19). Furthermore, treating the time-signal as an image allows to use network architectures specialized in image processing, which are more developed than its applications to signal processing. However, neglecting the signal nature of the data comes with disadvantages, as in wave-form delineation, the network output is not guaranteed to have a single value for each time, and requires post-processing to correct this. Future work would be to develop novel architectures taking advantage of the 1D/2D nature of the Doppler spectrograms.

When compared to other approaches in the literature, our methods compare favorably; although some solutions exist, they are partial and restricted to specific parts of the clinical analysis pipeline (37). Doppler view classification is difficult to address, and even though the approach by Gilbert et al. (12) achieves higher accuracy without requiring samples from multiple datasets, they benefited from certain advantages. Gilbert et al. had access to private vendor information (Doppler sample position), three times more data for model training, and dealt with data from the adult population. In contrast, our study involved training and testing models with data from two distinct contexts, encompassing different acquisition protocols, echo equipment, and the inter-observer variability as the two datasets were annotated by experts of different disciplines: FeDoC dataset was delineated by a paediatric cardiologist, whereas IMPACT was annotated by an obstetrician. Unlike the study presented by Gilbert et al. (12), this study did not benefit from having access to the sample probe position. To overcome the restricted access to it, its location was extracted with image processing. However, in contrast to Gilbert et al.'s (12) study, adding sample probe position did not improve the classifier's performance across both datasets (see Supplementary Figure S3). This discrepancy may be attributed to the lack of specificity in determining the exact sample probe position using image processing methods. The resulting binary mask, which contains the probe position, may take the shape of a bounding box or a dashed line, depending on the Doppler modality employed during image acquisition. This is illustrated in Supplementary Figures S13, S14, respectively. In addition, Doppler cursor position in the fetal population is less relevant than in adults, due to the variability of fetus’ position in mother's womb. The classifier achieved an accuracy of 89.2% in distinguishing between UA and MCA, while in the AoI vs. LVIO model, the accuracy stood at 67.3% in the IMPACT dataset. The second model's accuracy could potentially be enhanced, as demonstrated by an additional study detailed in the (Supplementary Figure S3). This study integrated IMPACT samples during model training, increasing classification accuracy by 25.5%, achieving a 92.8% accuracy when adding 251 samples per view to the model. The need for adding samples from the IMPACT dataset might be mostly due to differences during image acquisition across the datasets. The necessity for adequate training of sonographers to capture LVIO and AoI images was not fulfilled during the acquisition of the FeDoC dataset. It is possible that discrepancies in viewer settings may represent an additional source of variability between the two cohorts (38, 39). This is exemplified by the divergence between the sonographers in the IMPACT and FeDoC datasets, with the practice of zooming in on the B-mode region before capturing images, being exclusive to the IMPACT dataset.

**Figure 7:**
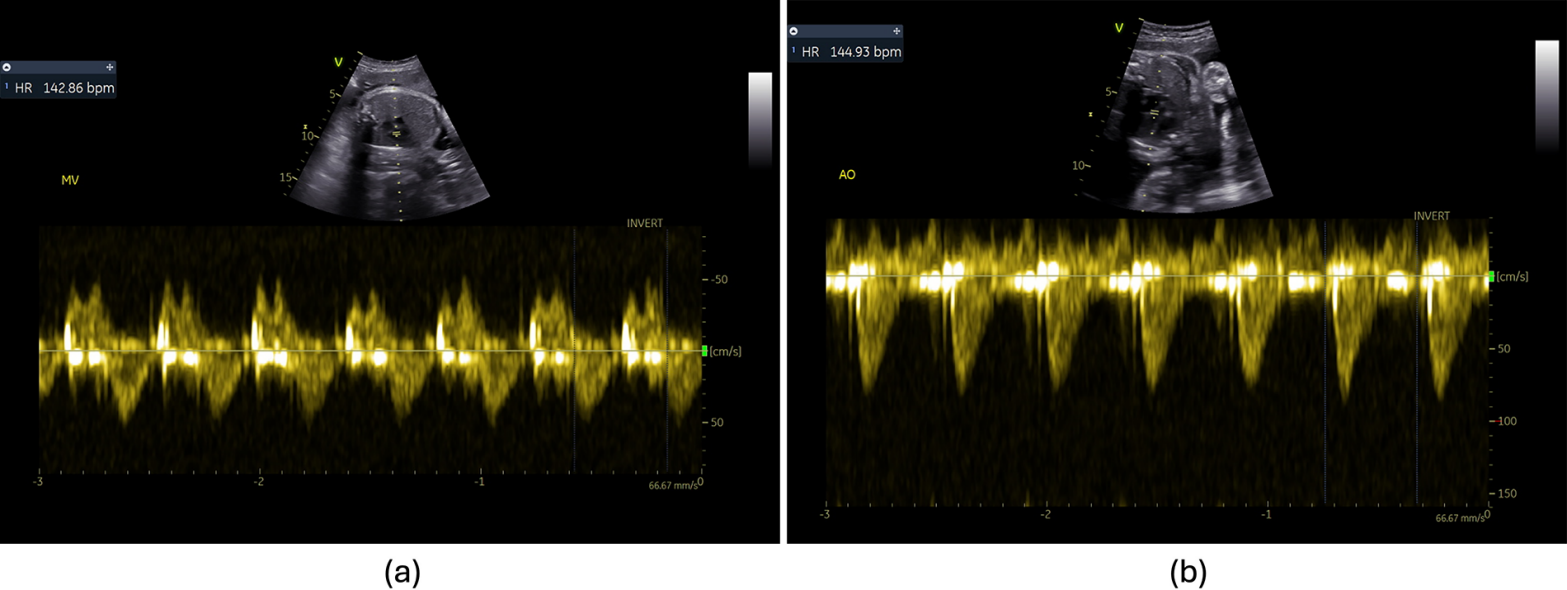
False positives of the LVIO confidence models used to detect misclassifications. **(a)** Image labelled as mitral valve and **(b)** image labelled as aortic valve.

In the medical domain, addressing misclassifications holds significant importance. In this context, we did not add an additional label to the developed DL-based classification models, considering the inherent variability observed in Doppler images and their spectra. With the aim of avoiding additional complexity in the DL models, our strategy focused on the creation of confidence models specific to each Doppler view and dataset. The developed models presented an overall accuracy over 85% across both datasets, consistently maintaining both the FNR and FPR below 15%. However, the LVIO confidence model's accuracy in the FeDoC dataset was notably lower, reaching only 27.8%. The selection of samples used to develop these confidence models was influenced by predictions made in earlier stages of the pipeline. Consequently, the training data for the LVIO confidence model might include Doppler views corresponding to heart valves, such as the MV and AV. Because the LVIO pattern combines elements from both left ventricular outflow and inflow, determining whether to retain or discard an image becomes challenging. Upon review, the identified FP images, classified by an expert as either MV or AV and predicted as LVIO by the *XGBoost* model, accounted for 1.7% of the test set. These images exhibited evident traces of both AV outflow and MV inflow within the Doppler region (see Figure 7).

Compared to the 25% inter-observer variability in measuring the maximal peak velocity reported by Vilkomerson et al. (8) for the location of relevant physiological events, our models demonstrate significantly reduced variability. Specifically, in MCA Doppler images, which presented the higher discrepancies between the ground-truth and the predicted values, we observed maximum MAPEs of 13.3 ± 4.7% and 10.8 ± 1.3%, for the FeDoC and IMPACT datasets, respectively. Despite the largest MAPEs in peak velocity detection are found in MCA images, the actual errors encountered in velocity magnitudes are quite small, being 4.5 ± 4.9 cm/s and 2.0 ± 3.0 cm/s. Additionally, in Zolgharni et al. (22), a 20% error was reported for the S peak, whereas our findings indicate a notably reduced error of less than 7%.

In assessing the timings of the S peak duration in LVIO images, our findings revealed an RMSE of 11.8 ± 7.3 ms (*n* = 380) and 14.8 ± 8.2 ms (*n* = 356) for FeDoC and IMPACT respectively. These values represent a smaller margin of error compared to the findings reported by Marzbanrad et al. *(*20), which indicated an RMSE of 38 ± 12 ms (*n* = 45). Larger bias values were found in our study compared to the Jevsikov et al. (18) in the detection of the E and A peaks in mitral inflow images. We report a bias of 2.7 ± 3.5 and 2.9 ± 4.8 cm/s, compared to their bias of 0.31 ± 2.00 cm/s for the detection of the E peak. In detecting the A peak, they found an error of 0.14 ± 1.54 cm/s, while our error was 2.9 ± 4.8 cm/s. Compared to our datasets, they used data from the same institution even if it was acquired by a time gap of three years, with a large dataset of 1,064 studies for training and 200 Doppler images for testing. The authors’ work is focused on the adult population, and it exclusively addresses the location of the mitral inflow peaks (*i.e.,* E and A peaks). In contrast, our approach employs a binary mask with distinct channels for the envelope and individual physiological events. Our detection of mitral valve inflow peaks is used to detect all relevant events on LVIO images, which include patterns from both aortic valve outflow and mitral valve inflow. This approach results in a greater number of points for detection and a higher level of complexity. Additionally, it is important to highlight that fetal Doppler presents greater challenges compared to adult Doppler, as it typically involves lower signal gain and higher variability, as well as a higher heart rate, further complicating the detection process.

Nevertheless, the variability observed in the extraction of Doppler measurements could be reduced by implementing additional image pre-processing techniques. One potential approach to enhance the consistency of the performance across models and datasets could be to ensure that the pixel-to-physical unit transformation is uniform across all images in both the training and the testing sets. This could involve resampling the images, in addition to the resizing already described in the pre-processing section.

The study presented here faced significant challenges stemming from the fundamental difference between the two datasets—FeDoC was a community-based observational study, while IMPACT was a hospital-based multi-arm clinical trial. Consequently, potential variations in imaging protocols, equipment, image characteristics, and quality between the two cohorts may arise, as well as differences in patient phenotypes. Such factors influence the deployment of models across both cohorts, complicating their ability to generalize across these distinct settings (40). Particularly, FeDoC was a community-based study (5), whereas IMPACT was a multi-arm clinical trial with strict inclusion criteria. To mitigate these challenges, several pre-processing steps aimed at standardizing input images, ensuring consistency, and maintaining uniform quality across diverse datasets were implemented. The pre-processing steps included the removal of burned-in annotations based on the detection of different vendor-specific colors, the resizing of the B-mode and spectral Doppler regions, and the intensity normalization of the image. Synthetic Doppler regions with a random number of cardiac cycles were created using image processing to overcome the difference in the duration of the Doppler spectrum between the two datasets during model training too. Additionally, the data augmentation techniques during model training were crucial to ensure the generalization and robustness of the hereby presented workflow across different ultrasound equipment and settings. The data augmentation techniques implemented included the synthetic generation of aliasing on the Doppler region, vertical and horizontal flipping of each of the regions, to account for differences in the acquisition angle and fetal positioning, and random cropping to mitigate the lack of zooming in on the B-mode region found in the FeDoC dataset. In the future, more complex data augmentation techniques presented here, when combined with more sophisticated AI-based solutions, such as Variational Autoencoders (VAEs) or Generative Adversarial Networks (GANs), could increase the performance of the models presented (41, 42).

In addition to the challenges associated with variations in image characteristics and data from different US equipment, another notable obstacle encountered was that the clinicians involved in the labelling and delineation of the Doppler images had different specialties: the exact definition of the velocity envelope varies depending on the disciplines and the guidelines followed by the clinical center. Addressing these challenges will be part of future work, focusing on consistent ground truth generation by the same expert and calculating inter-observer variability in Doppler envelope tracing. During model optimization, inter-observer variability could be used to ensure inferred Doppler indexes adhere to a maximum error threshold derived from such variability, penalizing those that surpass this predefined range. Moreover, while our work should be regarded as a proof of concept, future research is needed to validate the performance of the models that constitute the workflow presented in this study with additional external datasets to ensure their robustness and suitability for real-world deployment. Furthermore, the additional datasets could be employed to evaluate the performance of the waveform delineation models in comparison with the ground truth data derived from different experts, as was conducted in (43). This would allow us to investigate whether the proposed model optimization technique, based on interobserver variability, is able to reduce the divergence between the inferred results and the ground truth data with other datasets.

Considering all of the above, the approach proposed in this work presents several competitive advantages with respect to the state-of-the-art. The provided proof-of-concept AI solution covers with good performance all aspects of usual clinical care for feature extraction from Doppler images while being modular, so most advanced utilities can easily be incorporated into the system. However, automatization has to be carefully handled: a fully automated approach may lead to inaccurate measurements if unexpected issues arise, either due to the training set not being representative enough or bias in the generated ground-truth. Therefore, the AI-enabled workflow presented in this study has to be combined with the *TransCor Platform* providing clinicians with a full clinical decision support system where they can review the automatic classifications and waveform delineations and modify them, if necessary, before re-computing clinical measurements and use them in a report or a diagnosis. The presented AI-enabled workflow aimed at speeding up feature extraction from Doppler images, could be a preliminary step towards the creation of AI-driven models to be used for prenatal diagnosis. In addition, as future work, the *TransCor Platform* could be used not only to automate the analysis of feto-placental images, but also to train clinicians in the acquisition of high-quality data by creating a feedback-driven learning loop, in which clinicians can receive daily feedback on the quality of their acquired images by an expert in the field, allowing for continuous improvement of their techniques. Over time, variations in image quality can be used to train an AI model capable of recognizing suboptimal images by identifying patterns such as poor alignment, aliasing, or lack of gain. These models could then be incorporated into this workflow to extract measurements only from high quality images.

Several barriers must be addressed when considering the deployment of AI in resource-constrained healthcare settings. First, the lack of infrastructure and financial constraints pose significant challenges, as these settings often do not have the necessary resources to support the implementation of advanced technologies. Second, the absence of high-quality data can hinder AI performance, as reliable data is crucial for accurate decision-making and AI learning. Third, regulatory challenges also present obstacles, as stringent guidelines may slow down the adoption of AI in these environments. Lastly, integrating AI into existing clinical workflows can be difficult, as many healthcare systems in resource-limited settings are not designed to accommodate such technologies. On the other hand, several facilitators can promote the successful integration of AI in these settings. One key facilitator is demonstrating AI's effectiveness in improving clinical decision-making, which can help build trust among healthcare providers and encourage their engagement. This could be achieved by evaluating the performance of the workflow on several external datasets. The external datasets may contain images of varying quality, allowing the assessment of the efficacy of AI models across a range of image qualities. Another facilitator is the creation of affordable and scalable AI technologies, which would allow for cost-effective deployment in these settings, making AI solutions more accessible and sustainable.

In conclusion, the integration of AI into LMIC has the potential to be advantageous. Firstly, AI can provide medical expertise in areas where access to experienced healthcare professionals is limited. Secondly, AI can help to standardize assessments, reducing variability in diagnosis. Thirdly, AI can contribute to more efficient resource utilization and improve workflow efficiency, which is especially relevant in resource-constrained settings. Nevertheless, the implementation of decision support systems in a variety of healthcare settings necessitates meticulous consideration of several crucial elements. In addition to the technical considerations of hardware and software integration, it is essential to navigate the regulatory landscape that governs the use of AI in medicine (44–46). These regulations can vary significantly across regions and healthcare institutions. Future research could include the analysis of how existing solutions, such as Philips Intellispace Portal or Siemens’ eSie Measure software for automatic spectral tracing, successfully reached the market and were adopted by healthcare centers (47, 48).

## 5 Conclusions

It is our understanding that this work represents one of the earliest attempts to automate the end-to-end analysis of feto-placental Doppler images using AI (36). The included data augmentation and image pre-processing techniques were put in place to produce a performant and lightweight system. The good system performance and its completeness for automated feature extraction consolidates the proposed approach as a competitive solution for feto-placental Doppler image analysis. Using AI for this analysis has the potential to facilitate a more accurate and consistent assessment of fetal blood flow, heart function and placental health. This would enable the rapid processing of a large number of images and the early detection of fetal abnormalities. However, before the inclusion of the developed models into clinical practice, it is essential to consider the regulatory and ethical implications (44–46). In addition, the models presented in this study are designed for feature extraction from Doppler images only and are not intended to automate subsequent interpretation or decision-making (49). The responsibility for these crucial steps remains with clinicians, who will review the output generated by the AI models and make informed decisions based on their expertise.

## Data Availability

The imaging studies supporting the conclusions of this article will be made available by the authors, upon reasonable request.
