# Supplementary material for: AI-enabled workflow for automated classification and analysis of feto-placental Doppler images
Source: Front Digit Health. 2024 Oct 16;6:1455767. doi: 10.3389/fdgth.2024.1455767 (PMC11521966; doi:10.3389/fdgth.2024.1455767)
Supplement: Supplementary file 1 [file Table1.docx]

Supplementary Material

# Supplementary Figures


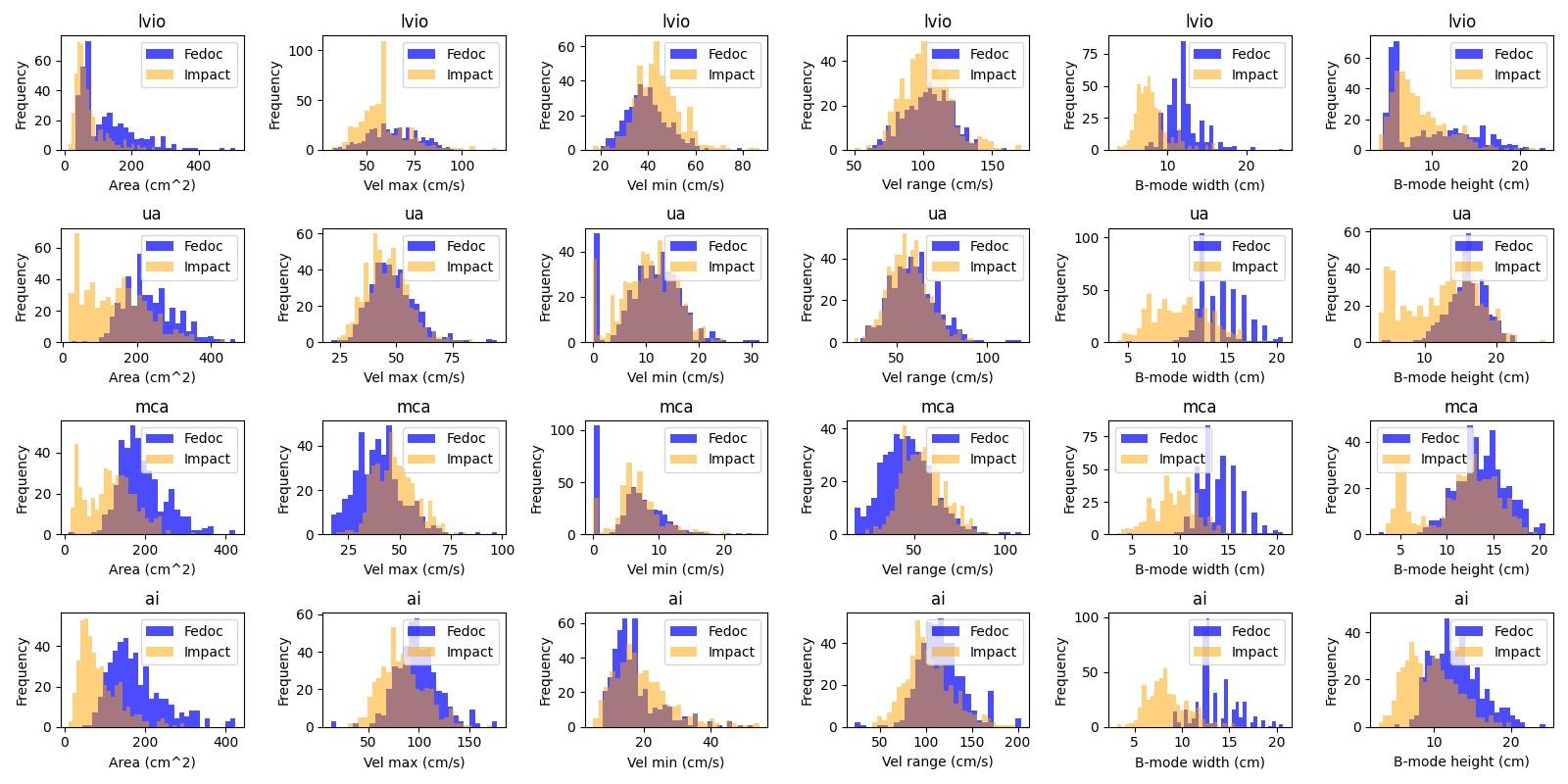


Figure S1: Distribution of metrics extracted from the Doppler spectra and B-mode in FeDoC and IMPACT datasets, using image processing techniques and public DICOM metadata information.


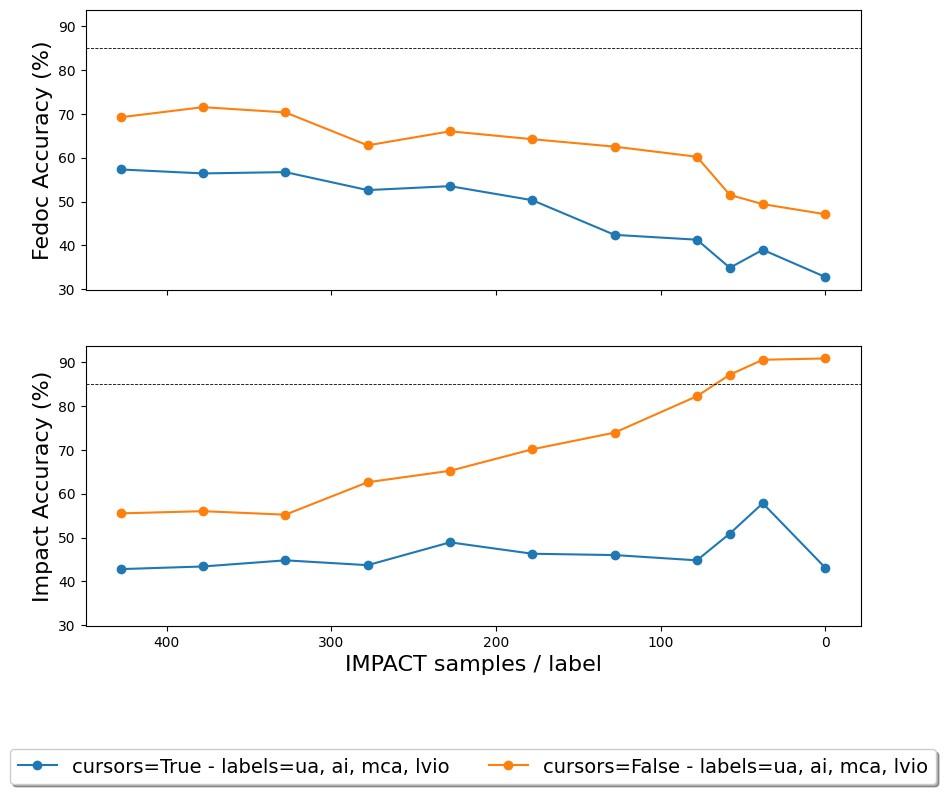


Figure S2: Doppler view classifiers accuracies for the umbilical artery (ua), aortic isthmus (ai), middle cerebral artery (mca) and left ventricular inflow outflow (lvio). Classifiers trained with variant number samples per view from the IMPACT dataset, with and without Doppler cursor position.


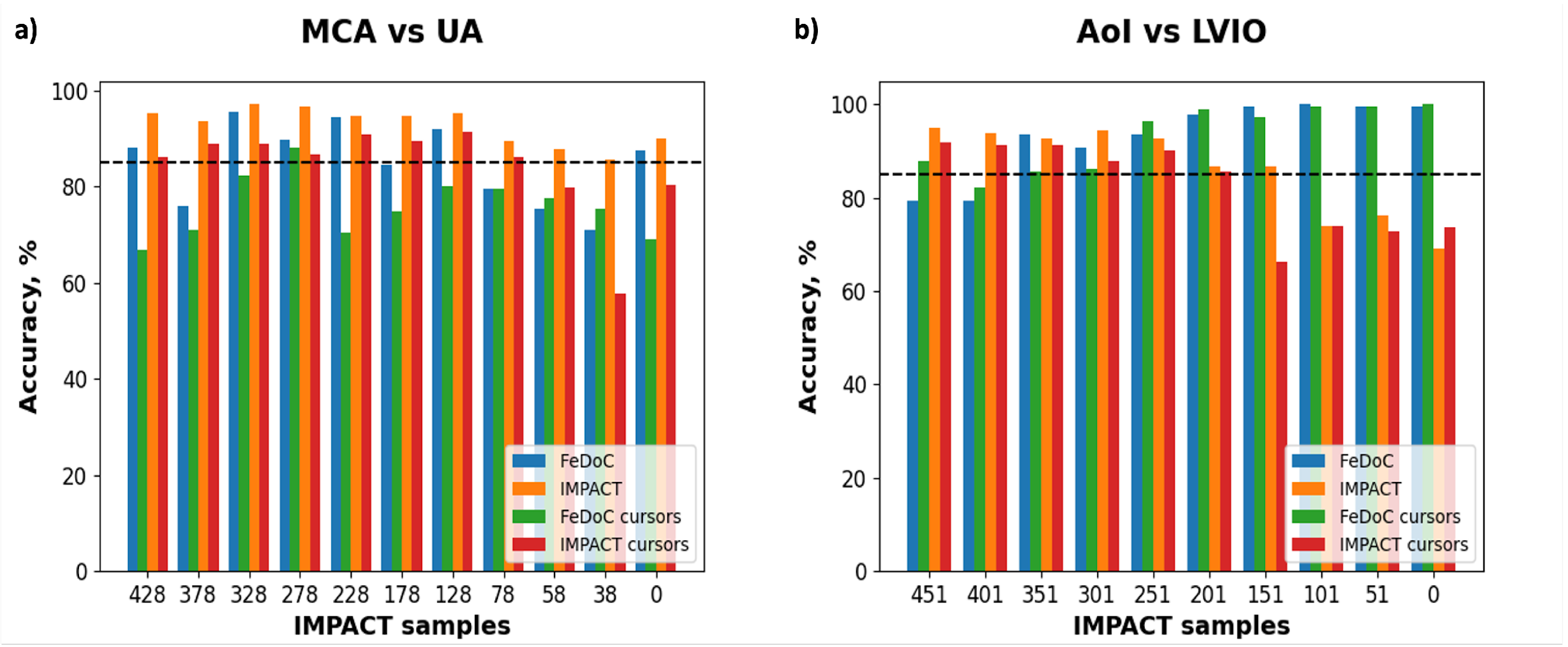


Figure S3: Reported accuracy scores with variant number of IMPACT samples per view, used to train the different DL-based classification models.


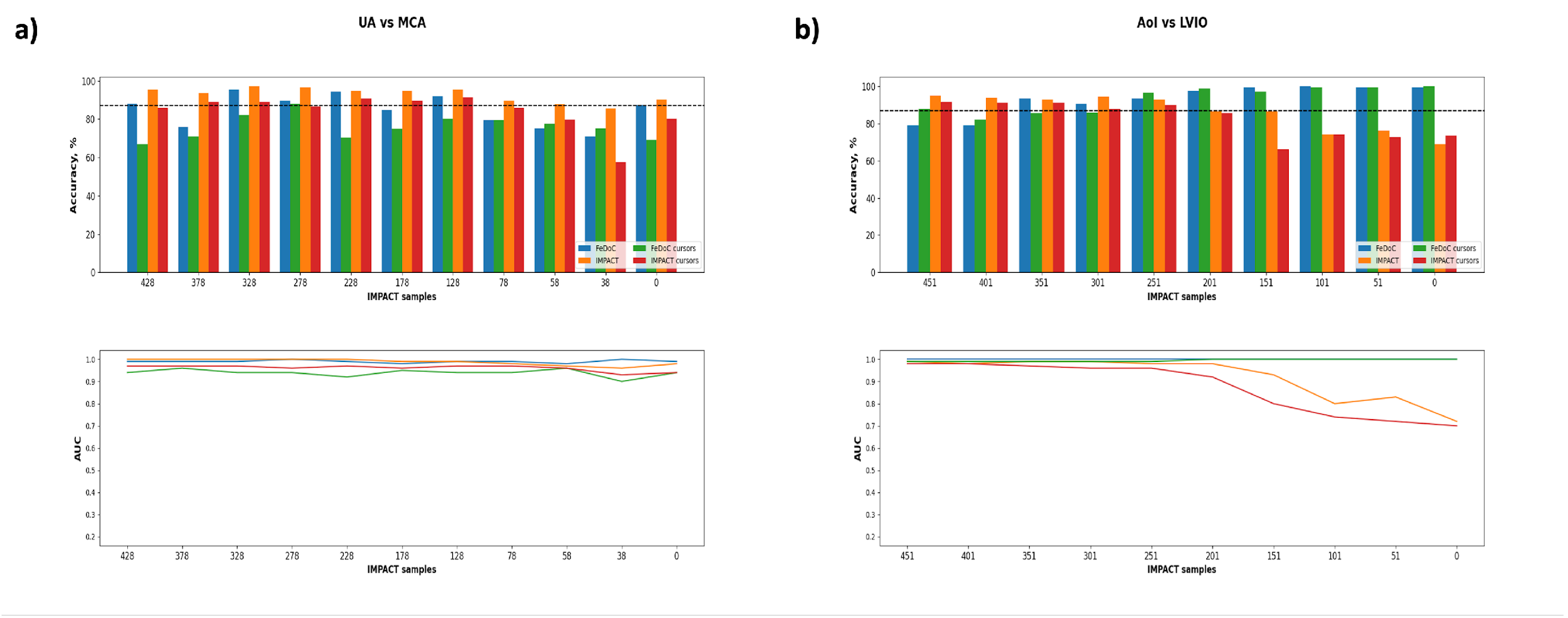


Figure S4: Reported accuracy and area under the curve (AUC) scores with variant number of IMPACT samples per view, used to train the different DL-based classification models.


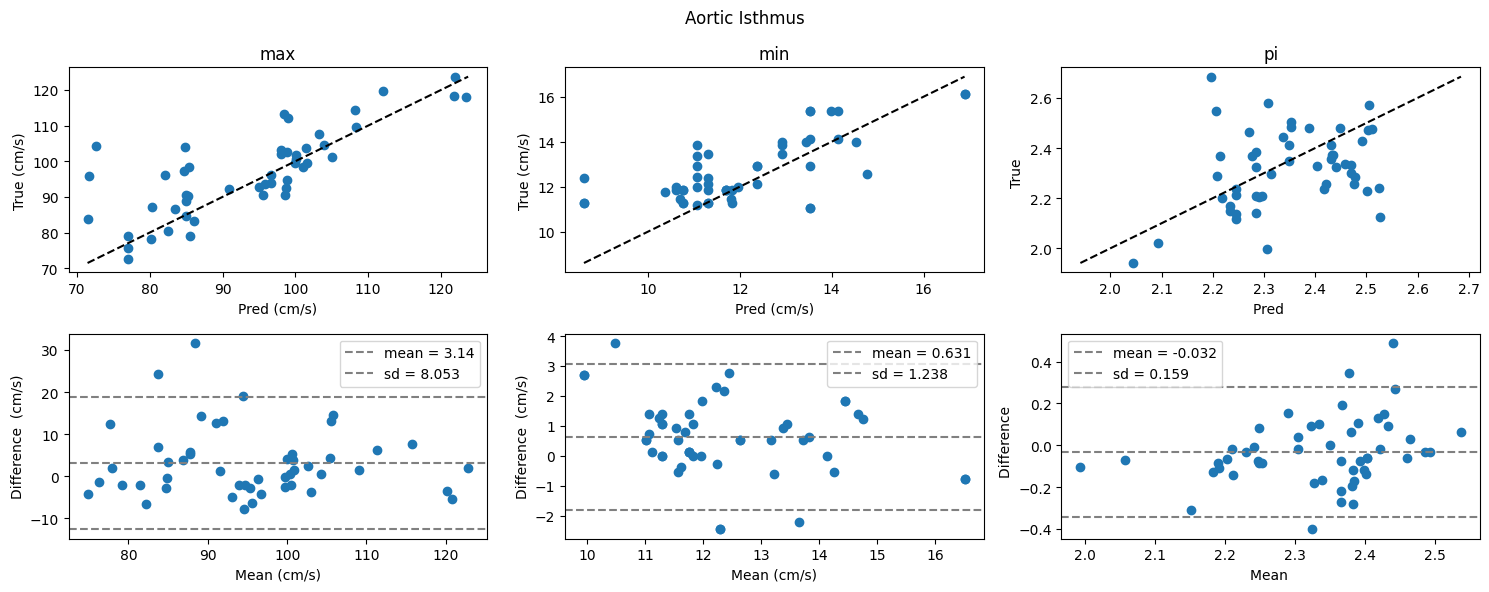
Figure S5: Bland-Altman graphs and comparing actual versus predicted Doppler indices (maximum (max) and minimum velocities (min), and pulsatility indexes (pi)) derived from aortic isthmus images from the FeDoC dataset.


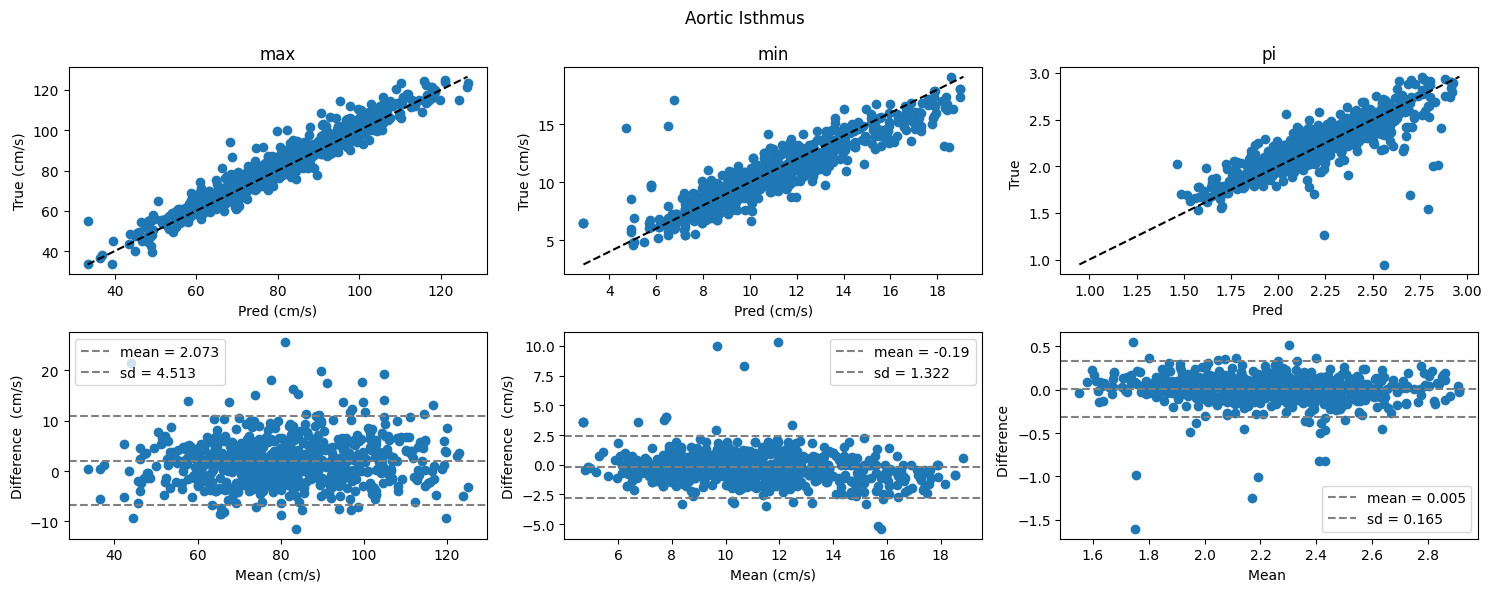


Figure S6: Bland-Altman graphs and comparing actual versus predicted Doppler indices (maximum (max) and minimum velocities (min), and pulsatility indexes (pi)) derived from aortic isthmus images from the IMPACT dataset.


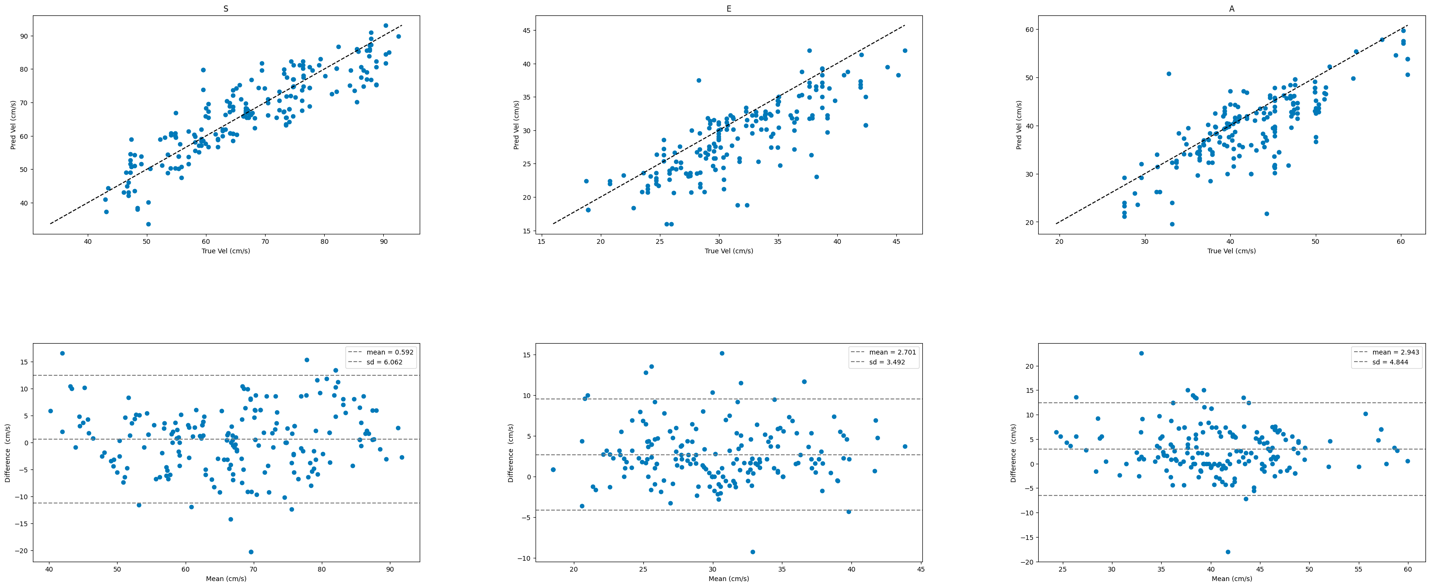


Figure S7: Bland-Altman graphs and comparing actual versus predicted Doppler indices (S, E and A peaks) derived from left ventricular inflow outflow images from the FeDoC dataset.


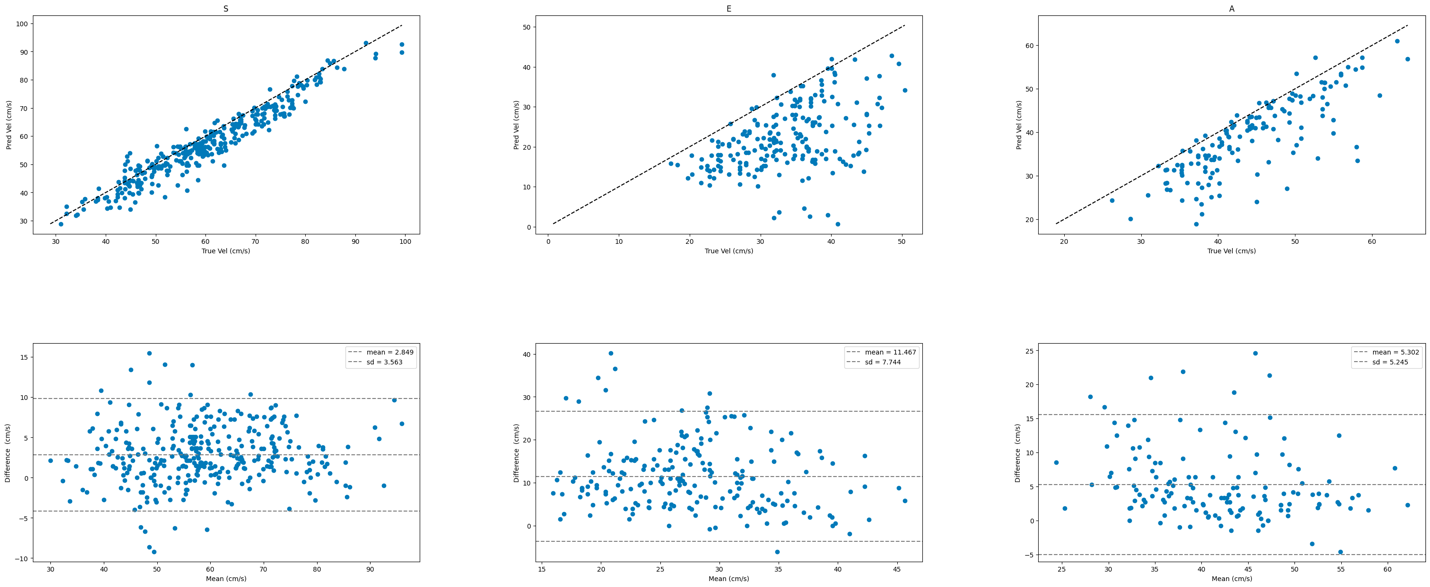


Figure S8: Bland-Altman graphs and comparing actual versus predicted Doppler indices (S, E and A peaks) derived from left ventricular inflow outflow images from the IMPACT dataset.


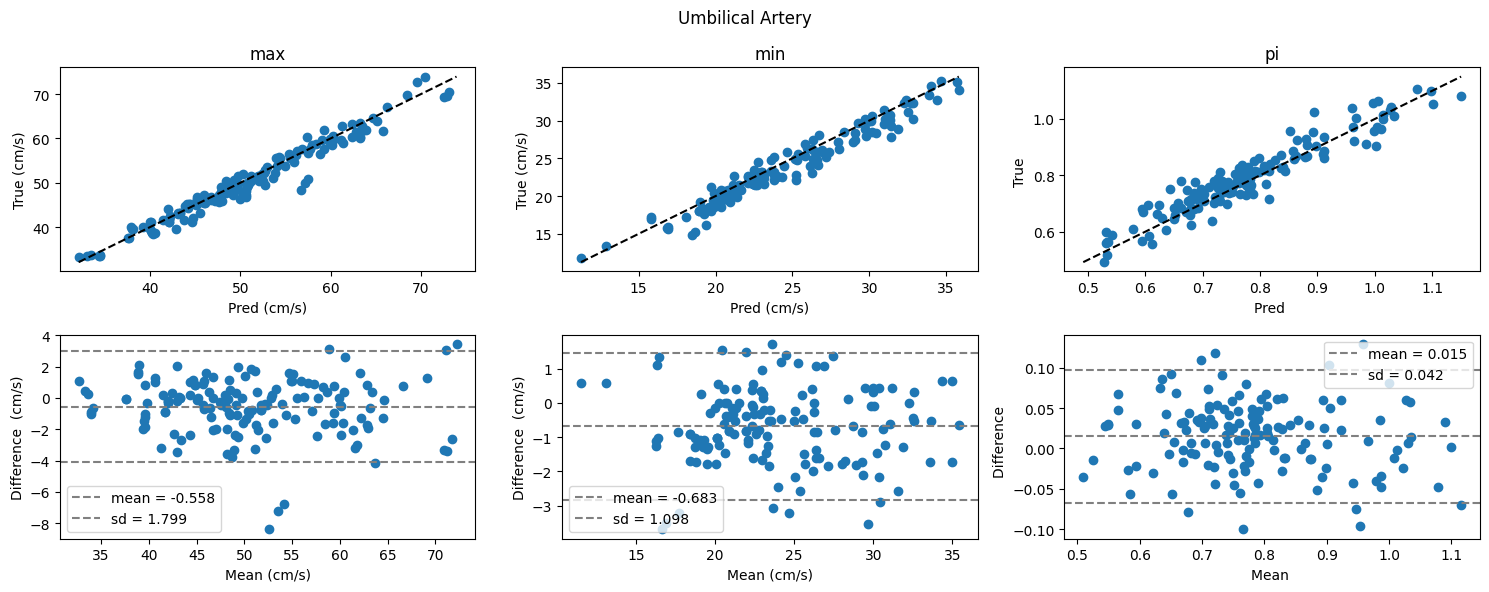


Figure S9: Bland-Altman graphs and comparing actual versus predicted Doppler indices (maximum (max) and minimum velocities (min), and pulsatility indexes (pi)) derived from umbilical artery images from the FeDoC dataset.


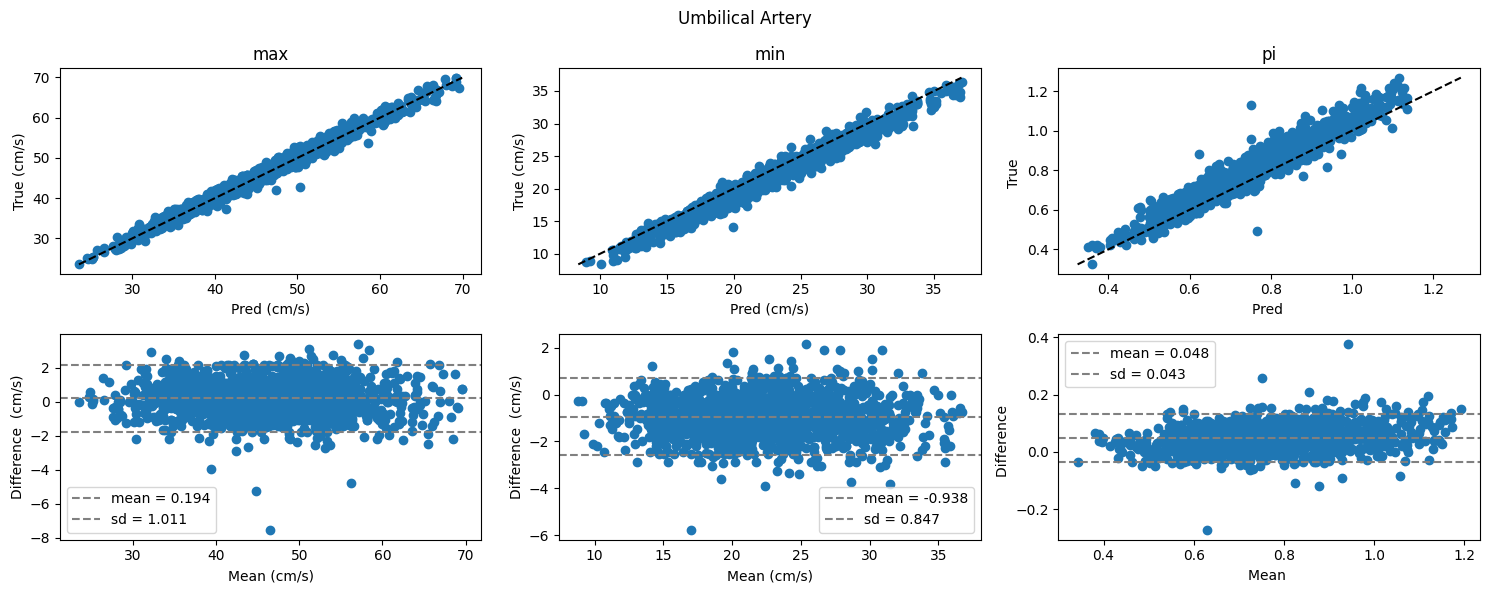


Figure S10: Bland-Altman graphs and comparing actual versus predicted Doppler indices (maximum (max) and minimum velocities (min), and pulsatility indexes (pi)) derived from umbilical artery images from the IMPACT dataset.


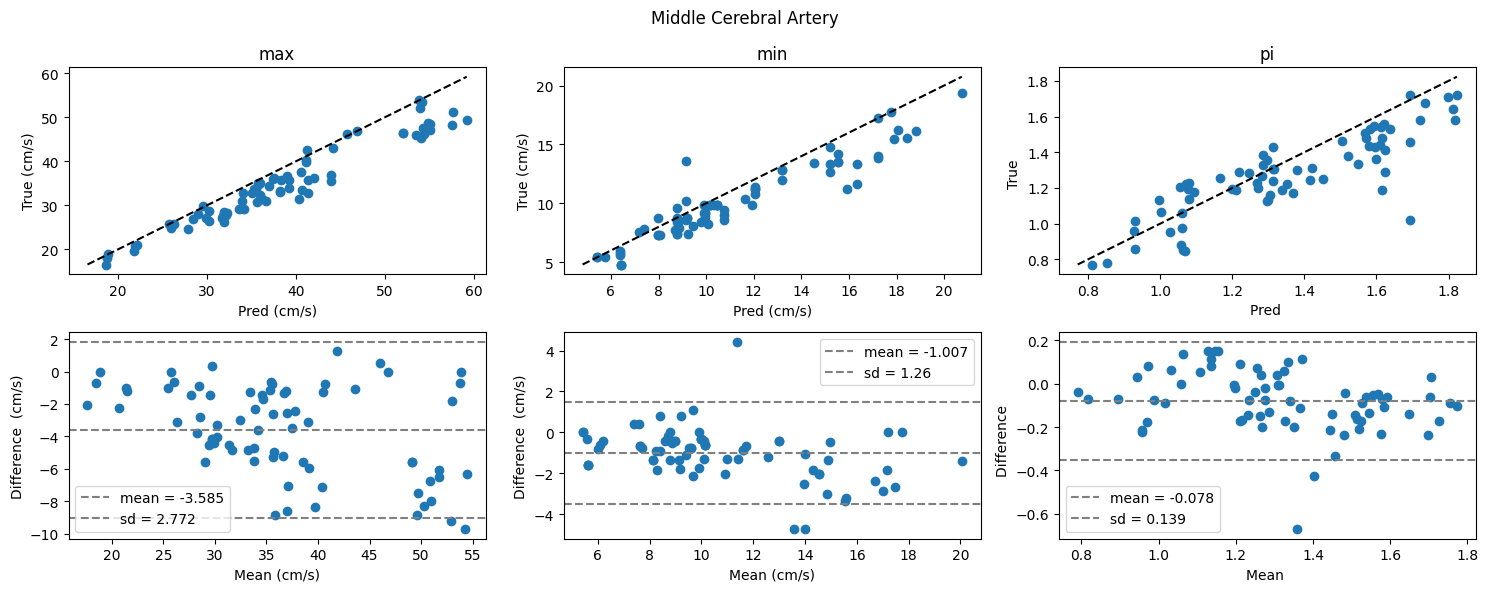


Figure S11: Bland-Altman graphs and comparing actual versus predicted Doppler indices (maximum (max) and minimum velocities (min), and pulsatility indexes (pi)) derived from middle cerebral artery images from the FeDoC dataset.


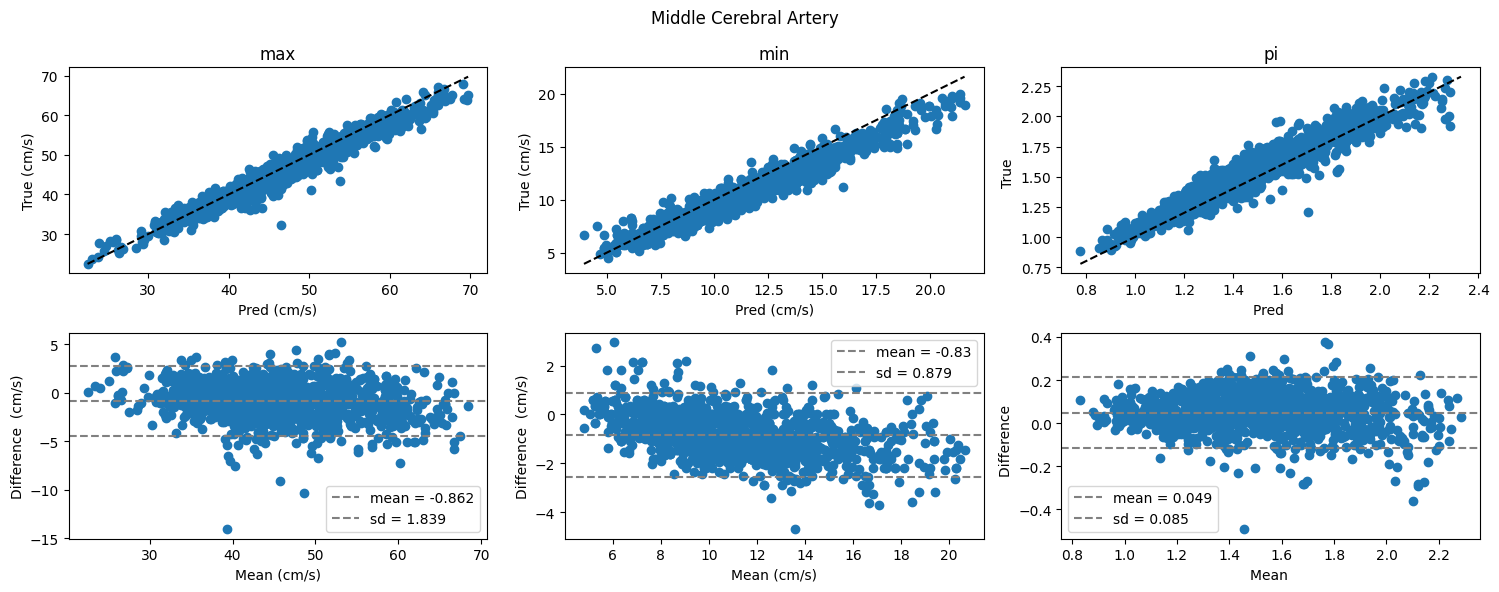


Figure S12: Bland-Altman graphs and comparing actual versus predicted Doppler indices (maximum (max) and minimum velocities (min), and pulsatility indexes (pi)) derived from middle cerebral artery images from the IMPACT dataset.

**
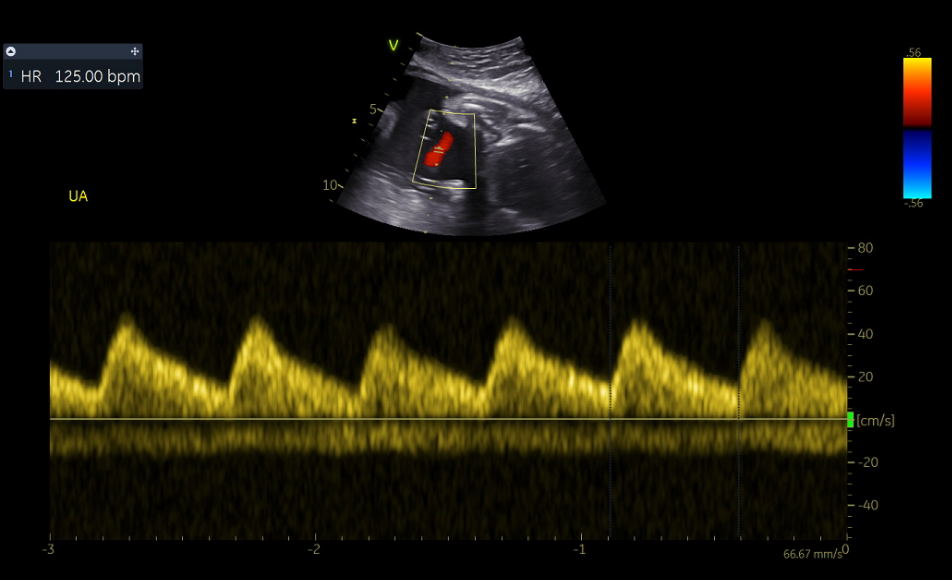
**

Figure S13: Doppler image corresponding to an umbilical artery acquired using pulse waved Doppler (PWD), where the B-mode cursor is illustrated with a bounding box.

**
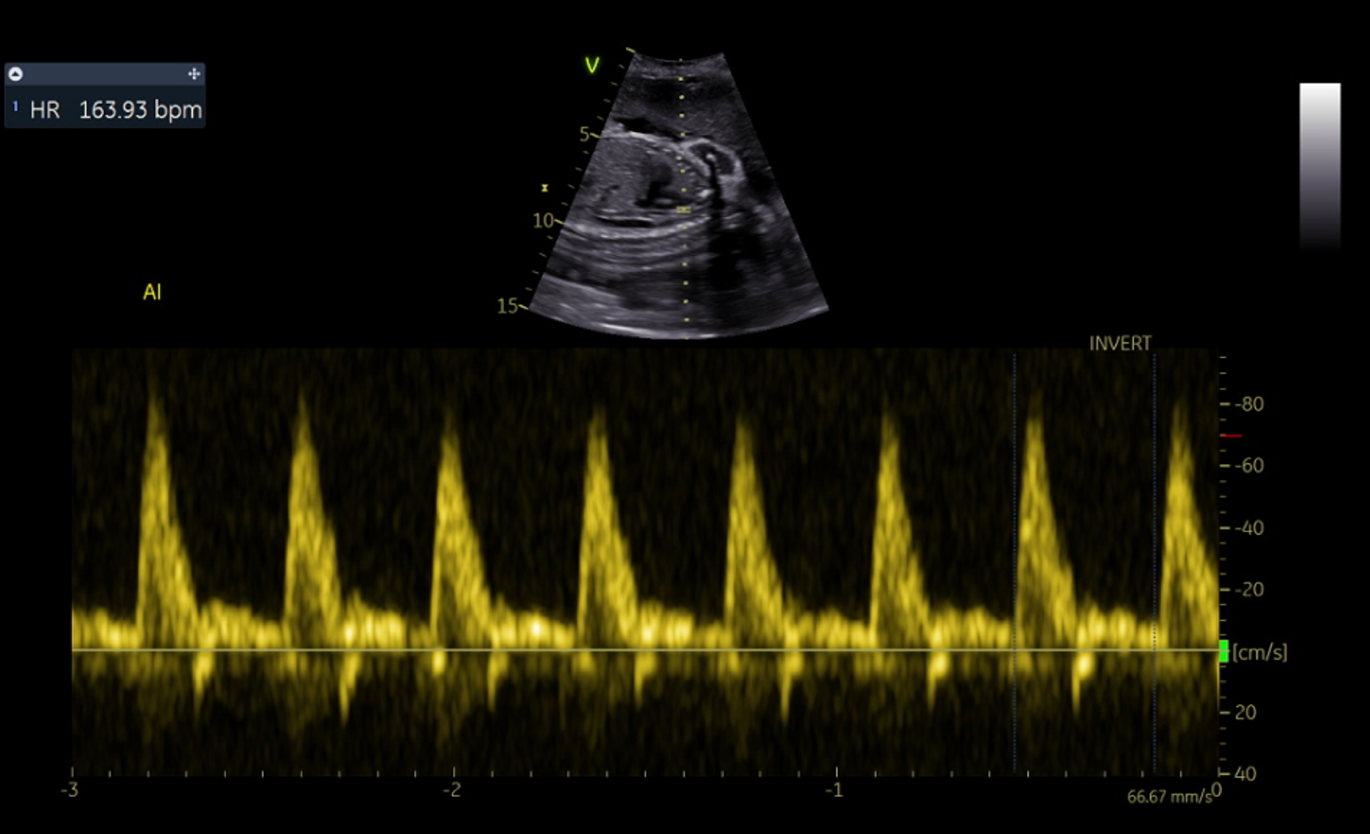
**

Figure S14: Doppler image corresponding to an aortic isthmus (AoI) acquired using continuous Doppler (CW), where the B-mode cursor is represented by a dashed line.

**
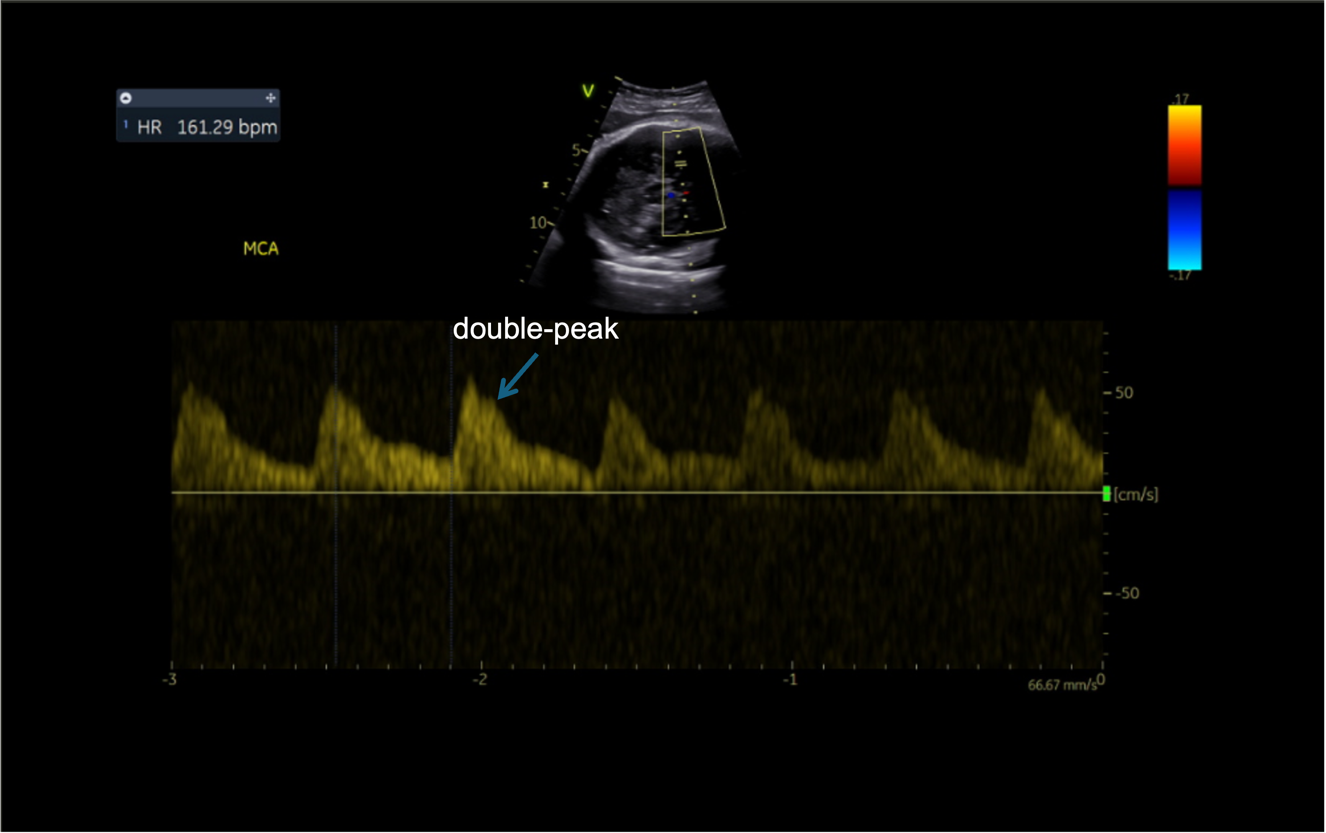
**

Figure S15: Doppler image corresponding to a middle cerebral artery (MCA) that contains a double peak on the wave.

# Supplementary tables

| **Label** | **Duration, s** | **FeDoC** | **IMPACT** |
| --- | --- | --- | --- |
| **MCA** | **Mean** | 3.0s | 2.96s |
|  | **Max** | 4.0s | 6.07s |
|  | **Min** | 2.0s | 0.96s |
|  | **Samples** | 360 | 631 |
| **UA** | **Mean** | 3.0s | 2.84s |
|  | **Max** | 3.87s | 6.07s |
|  | **Min** | 2.50s | 0.96s |
|  | **Samples** | 352 | 627 |
| **AoI** | **Mean** | 3.01s | 2.95s |
|  | **Max** | 4.0s | 6.07s |
|  | **Min** | 2.0s | 0.96s |
|  | **Samples** | 377 | 612 |
| **LVIO** | **Mean** | 3.0s | 1.34s |
|  | **Max** | 3.0s | 3.82s |
|  | **Min** | 2.96s | 0.96s |
|  | **Samples** | 297 | 410 |

Table S1: Table showing the mean, maximum and minimum duration (in seconds) of the Doppler spectrograms collected from the two datasets.
